# Microbiota and Metabolite Profiling of Spoiled Spanish-Style Green Table Olives

**DOI:** 10.3390/metabo8040073

**Published:** 2018-10-31

**Authors:** Antonio de Castro, Antonio Higinio Sánchez, Antonio López-López, Amparo Cortés-Delgado, Eduardo Medina, Alfredo Montaño

**Affiliations:** Food Biotechnology Department, Instituto de la Grasa-CSIC, Utrera road, km 1, 41013 Seville, Spain; adcastro@ig.csic.es (A.d.C.); ahiginio@ig.csic.es (A.H.S.); all@cica.es (A.L.-L.); acortes@cica.es (A.C.-D.); emedina@ig.csic.es (E.M.)

**Keywords:** green table olives, spoilage, microbial community, DNA sequencing, SPME-GC-MS, metabolite composition, *Cardiobacteriaceace*, *Ruminococcus*

## Abstract

The aim of the present study was to assess the malodorous spoilages of Spanish-style green table olives through microbial and metabolite composition using current measuring techniques (e.g., high-throughput DNA sequencing, headspace solid-phase microextraction combined with gas chromatography-mass spectrometry). Under different alkaline and washing conditions, the spoilage fermentations were reproduced with Gordal and Manzanilla olive cultivars using a low salt concentration (71 g L^−1^ NaCl) in the initial brine. The degradation of lactic acid and significant increases in volatile fatty acids and phenols were found in all the spoiled samples in comparison with the unspoiled control samples. According to high-throughput DNA sequencing, *Cardiobacteriaceae* and *Ruminococcus* were the dominant bacteria in the spoiled samples. PLS regression and Pearson’s correlation coefficient analyses revealed positive and negative correlations among microbial communities, metabolites, and sensory spoilage descriptors. Notably, the “zapatera” descriptor was significantly associated with *Propionibacterium*, which was positively correlated with acetic acid, propionic acid, succinic acid, and methyl propanoate; while the “butyric” descriptor exhibited a significant positive relationship with the genus *Ruminococcus*, which gave an almost significant correlation with propionic and butyric acids.

## 1. Introduction

Spanish-style green olives are one the most highly-valued fermented vegetables. Spain is the main producer of different types of table olives with around 550,000 tons produced annually [1]. Among them, approximately 250,000 tons correspond to the green Spanish-style, whose processing is characterized by an initial alkaline treatment with 20–35 g L^−1^ sodium hydroxide (lye) for several hours; then a wash step with tap water is carried out in order to remove excess alkali, and finally the olives are covered with a 90–110 g L^−1^ sodium chloride brine where microbial fermentation takes place [2]. Normally, the fermentation is carried out by environmental lactic acid bacteria and yeasts without the addition of starters [3]. The table olive industry occasionally reports the spoilage of fermented green olives associated with increases in brine pH and unpleasant odors. Olives may suffer different kinds of microbial spoilage if the microbial population is not well controlled during the different phases of fermentation [4]. Three different types of malodorous spoilage have been recognized in this product: zapatera, butyric, and putrid. The flavor of olives affected by the zapatera spoilage is hard to describe but is clearly abnormal and very distinctive. Although the characteristic off-odor of the zapatera samples is different from that of butyric or putrid fermentations (the butyric deterioration is reminiscent of the smell of rancid butter, while the putrid type is reminiscent of the smell of decomposing organic matter), there is sometimes confusion over the term “zapatera”, with a tendency to classify most olives with abnormal flavor as such. Since the finding of cyclohexanoic acid in appreciable amounts in zapatera olives [5], it is recognized that this acid is mainly responsible for the typical off-odor of this spoilage. In fact, aqueous solutions of cyclohexanoic acid are used as reference materials for taster and panel leader training in the sensory assessment of table olives [6].

The volatile metabolites produced by the microorganisms involved in olive spoilage are responsible for the off-odor. These microorganisms have been found to belong to the genus *Clostridium* in all three of the above-mentioned types of spoilage, although *Propionibacterium* can also be prominent, especially in the case of zapatera spoilage [4]. Although butyric and cyclohexanoic acids are the main volatile metabolites responsible for butyric and zapatera spoilages, respectively, other metabolites could be associated with each type of spoilage. Propionic, 3-methylbutyric, valeric, and caproic acids have been found at concentrations above their odor threshold in the case of zapatera olives [5]. However, other volatile compounds, which may be closely related to spoilage, have not been identified to date. A deeper knowledge of the microbial and metabolite compositions of spoiled table olives is necessary for a better characterization of each type of spoilage.

All the above-mentioned investigations on microbial spoilage in table olives were carried out decades ago. DNA-based methods are nowadays the most commonly applied techniques for microbiota identification in fermented vegetables (including table olives). Among them, high-throughput sequencing (HTS) has been used to study different food matrices [7]. These techniques confer a more comprehensive identification of the different taxa than the classical culture-dependent methods. However, molecular methods have hardly been used in spoiled table olives [8]. Regarding volatile metabolites, headspace solid-phase microextraction (HS-SPME) combined with gas chromatography-mass spectrometry (GC-MS) is currently one of the most popular techniques for the analysis of volatile compounds in food [9]. This technique has recently been used by our group to study the volatile composition of Spanish-style green table olives [10,11,12,13,14,15], but so far it has not been applied to spoiled table olives.

The main objectives of this research were (1) to investigate the microbial and chemical composition of spoiled Spanish-style green olives, and (2) to evaluate the relationships between microbial communities, metabolites, and sensory spoilage descriptors. 

## 2. Results and Discussion

### 2.1. Physicochemical Characteristics of Brine Samples

**Figure 1:**
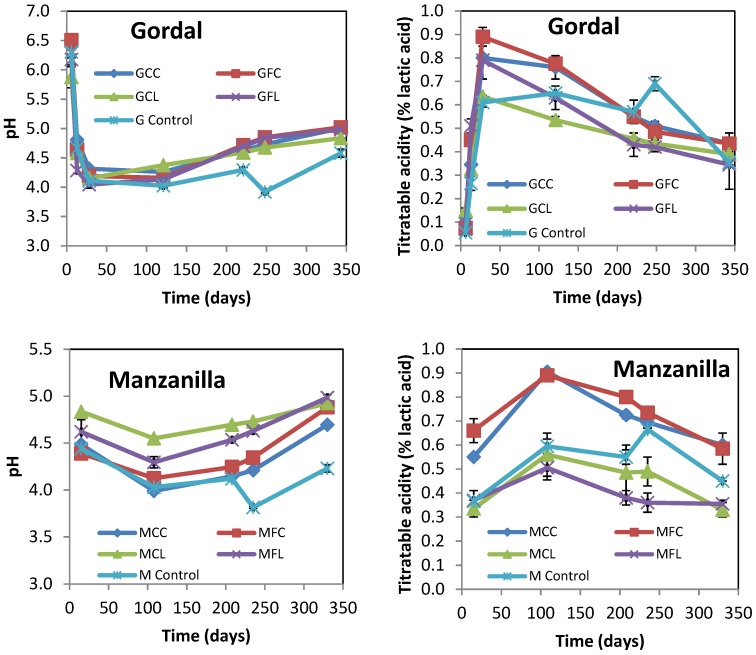
The changes in the pH and titratable acidity of Gordal and Manzanilla samples during fermentation. The points are the means of duplicate fermentations. Error bars show the range of the data (n = 2). Where error bars are not visible, the values were within the range of the symbols. See Table 5 for the meanings of samples abbreviations.

The evolution of pH and the titratable acidity of brine samples from Gordal and Manzanilla cultivars at up to 11 months of brining are shown in Figure 1. 

The initial increase in titratable acidity due to lactic acid fermentation occurred faster in the Gordal samples compared to the Manzanilla samples. In all the samples but the control ones, the titratable acidity reached its maximum value during the fermentation step, and progressively decreased afterward according to the measured pH increase. The final values for pH ranged between 4.66 and 5.10. It is worth noting that in the control samples from both cultivars titratable acidity increased with a concomitant decreased in pH as a result of adding lactic acid after 8 months of brining, but then the titratable acidity decreased during the post-fermentation stage (Figure 1). This could be attributed to oxidative yeasts growing on the surface of brine, which partially metabolized the lactic and acetic acids (Figure S1). The final physicochemical characteristics of all samples are shown in Table 1. The different values of combined acidity and total phenols were indicative of the different alkaline treatments and washing steps applied. All samples with low salt concentration (35–39 g L^−1^ NaCl in brine) were detected by smell as spoiled to a greater or lesser extent. On the contrary, all the control brine samples (with 87–88 g L^−1^ NaCl) had normal odors. The abnormal odors of samples with low salt were confirmed by a quantitative descriptive analysis, as discussed later. 

### 2.2. Microbiota in Brine Samples

**Figure 2:**
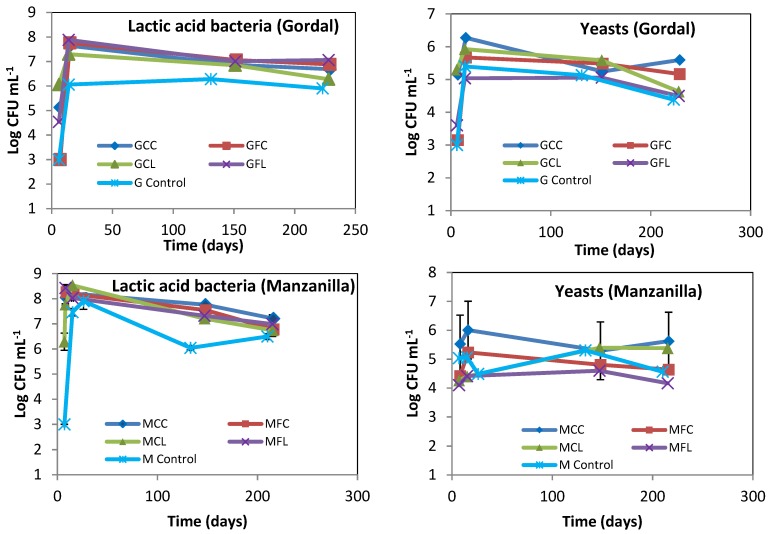
The microbial count of lactic acid bacteria and yeasts during fermentation. The points are the means of duplicate fermenters. Where the error bars (range of data) are not visible, the determinations were within the symbols on the graph. See Table 5 for the meanings of samples abbreviations.

The lactic acid bacteria and yeast populations obtained by plate counting are presented in Figure 2. The lactic acid bacteria grew rapidly and high numbers were present from the first few days, which explain the rapid acidity increase and pH decrease, as shown in Figure 1. The LAB populations in the Gordal and Manzanilla controls seemed to be lower than in the other treatments, probably as a logical consequence of the importance that salt concentration has on bacterial growth. With regard to yeasts, it is important to highlight that their number were below those of the LAB; and this is an indication that the fermentation process developed normally.

Apart from the culture-dependent analysis carried out throughout the fermentation process, microbial DNA was extracted and analyzed at the post-fermentation stage once spoilage was detected. The high throughput sequencing of the 16S- and ITS-PCR products generated a mean of 71,189.33 and 46,906.61 high-quality sequences per sample with an average length of 459 and 495 bp, respectively (Tables S1 and S2). Across all taxa, a total of 3 families and 10 genera were identified as predominant bacterial Operational Taxonomic Units (OTU relative abundance >0.1%). Likewise, 1 family and 5 species were identified for ITS. The microbial community was also analyzed using a diversity estimator (Shannon, Simpson, and Chao1) (Tables S1 and S2). Manzanilla samples showed a higher diversity than Gordal for ITS amplicons and no clear differences were found for 16S samples.

**Figure 3:**
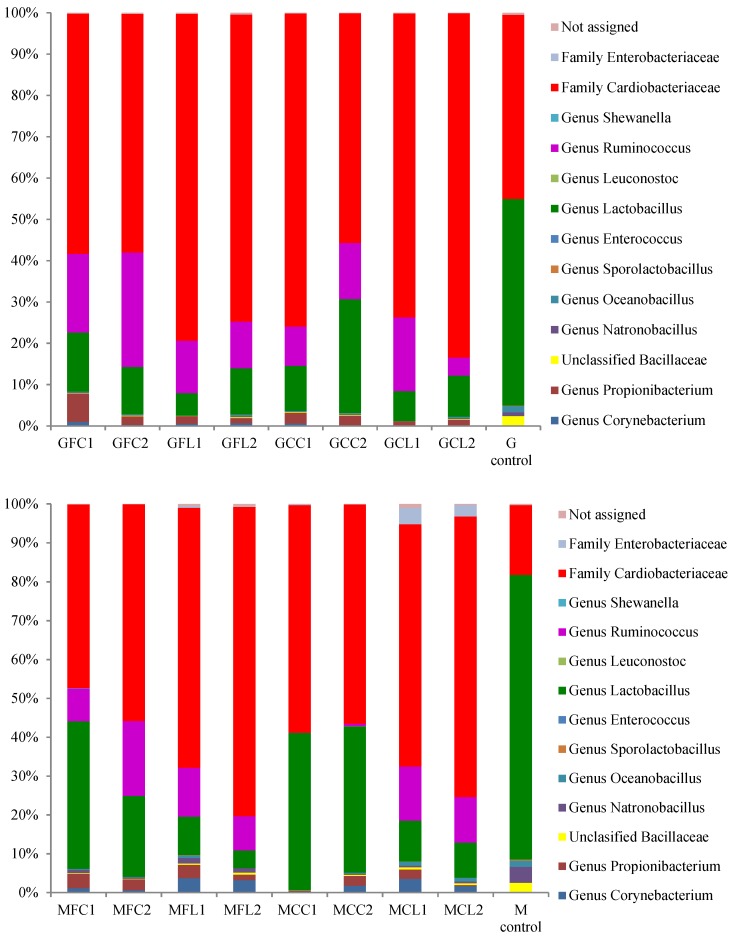
The relative abundance (%) of the bacterial genera or family obtained by the pyrosequencing analysis at the post-fermentation stage. The results of the G and M controls correspond to a blend of both duplicates. See Table 5 for the meanings of the samples abbreviations.

**Figure 4:**
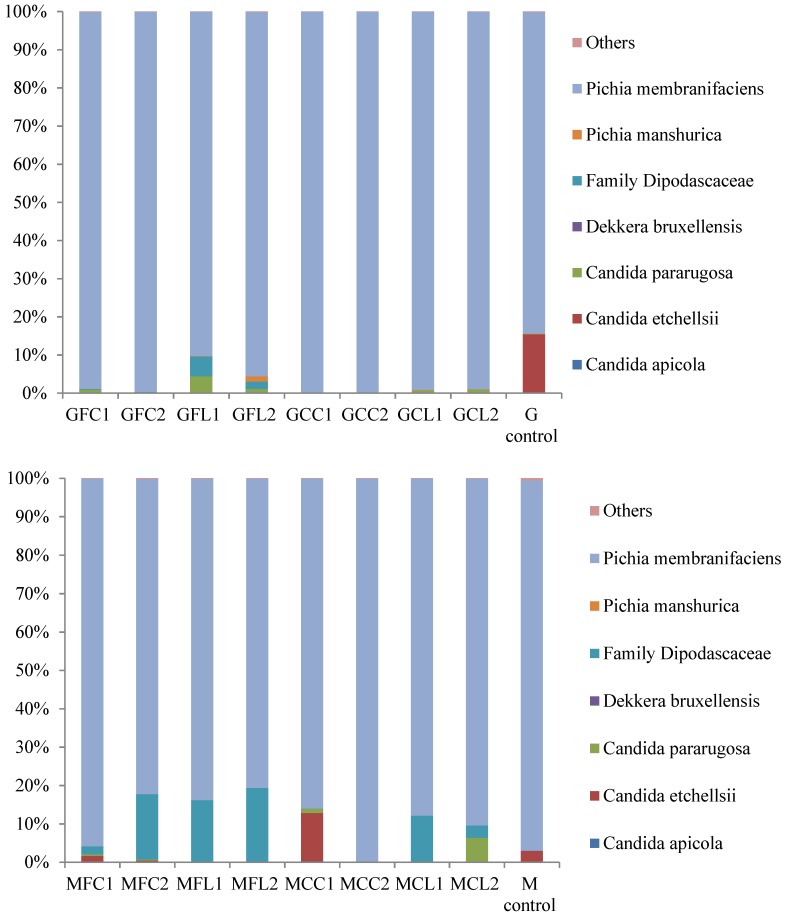
The relative abundance (%) of the fungal species or family obtained by the pyrosequencing analysis at the post-fermentation stage. The results of the G and M controls correspond to a blend of both duplicates. See Table 5 for the meanings of the samples abbreviations.

The main taxa found at the end of the post-fermentation stage are displayed in Figure 3 for bacteria and Figure 4 for yeasts. 

The most striking result in relation to bacteria is the very high relative abundance of sequences belonging to the *Cardiobacteriaceae* family, initially and tentatively allocated to the genus *Suttonella*, which were predominant in all the vessels except in both the Gordal and Manzanilla controls. These Gram-negative *Gammaproteobacteria* have been mainly related to the upper respiratory tract of human and animal samples, both mammals and birds, manifesting pathogenicity in some cases [16]. However, they seem to be normal microorganisms in dolphins [17,18], and penguin stomach contents [19] and even in seawater [20]. More unexpectedly, *Suttonella* sp. has been previously found in defective table olives [8] and in spoiled fermented cucumbers [21]. A suggested origin of some species isolated from table olive brines is the salt used for preparing fermented vegetables, which is usually of marine origin [22]. In addition to *Cardiobacteriaceae*, *Lactobacillus* was the main genus found in the controls and its presence was also considerable in the other vessels. Lactic acid bacteria in general and specifically *Lactobacillus pentosus* are responsible for a suitable fermentation of Spanish-style green olives [3]. The other important genus with high relative abundance in the spoiled samples, with the exception of the MCC treatment, was *Ruminococcus*. The absence of this genus in MCC1 and MCC2 could be attributed to their relatively high phenolic content (Table 1), which inhibited bacterial growth. In addition, *Ruminococcus* was absent in the controls, indicating that this genus may be sensitive to the high levels of NaCl in these samples. *Ruminococcus* has been found in the rumen of many different species [23] and is present in the human gut microbiota as well [24]. To our knowledge, it has not been related to table olives before the present study, although it has been identified in Italian fermented sausages [25] and in a Chinese fermented grain product [26]. In relation to the yeast populations (Figure 4), *Pichia membranifaciens* stood out as the most abundant species in all the vessels. In fact, it accounted for more than 90% of all the sequences in almost all the Gordal samples and half of the Manzanilla samples. This species has been isolated from practically all table olive preparations [27], and its capacity to assimilate lactic acid is well-known [28]. Other significant yeast taxa were the species *Candida etchellsii* and *C. pararugosa*, and the family *Dipodascaceae*, which has been recently found with the highest relative abundance in samples of industrial olives darkened by oxidation [29].

### 2.3. Metabolite Composition in Brine Samples

The metabolite analysis in brine at the end of the post-fermentation stage for samples from Gordal and Manzanilla cultivars are shown in Table 2 and Table 3, respectively. 

All metabolites, with the exception of lactic and succinic acids, were volatile compounds and grouped into different chemical classes: acids, alcohols, aldehydes, esters, phenols, terpenes, and other compounds. Acetic and propionic acids were the major volatile compounds in all the samples, both spoiled and control, with levels well above their reported odor thresholds in water (acetic acid, threshold 26 mg L^−1^; propionic acid, threshold 2 mg L^−1^) [30,31]. Lactic acid was not detected in any of the spoiled brines, with the exception of MCC1, which contained 3.9 g L^−1^. Lactic acid is the main end-product of lactic acid fermentation and is the major metabolite in “normal” Spanish-style green table olives [32]. The degradation of lactic acid and concomitant formation of C_2_-C_6_ volatile acids have been reported as a result of zapatera spoilage in olives [5]. All the spoiled samples had significantly higher contents of volatile acids and phenols compared to the control samples (Figure S2). High amounts of volatile phenols were also found in olive oils with sensory defects, as a result of the activity of microorganisms [33]. Two volatile compounds, which were present in the spoiled samples, but not found in the controls, were benzyl propanoate and *o*-guaiacol. However, these compounds should not be considered as potential spoilage marker candidates because both compounds have been found in normal (unspoiled) samples of Spanish-style green table olives in previous studies [13,14]. 

**Figure 5:**
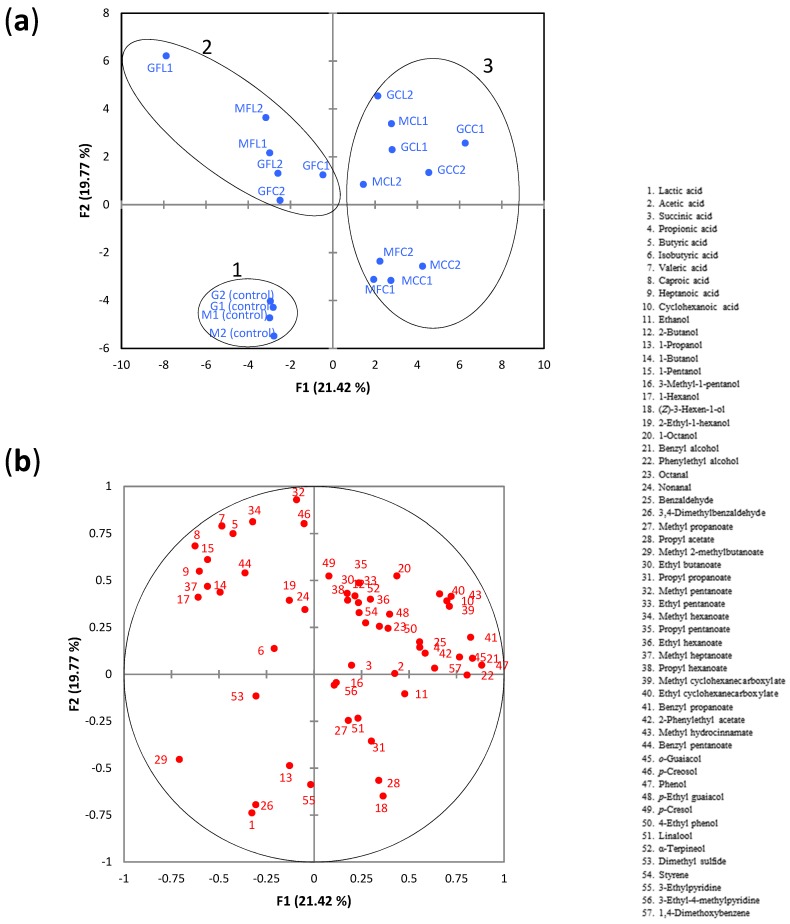
(**a**) The PCA score plot of metabolite data, (**b**) the PCA loading plot of metabolite data. The 3 groups identified by agglomerative hierarchical cluster (AHC) analysis are highlighted by ellipses. See Table 5 for the meanings of samples abbreviations.

When the whole dataset was considered, PCA showed a clear separation between the unspoiled control samples and spoiled samples, which were located in different quadrants (Figure 5a). 

Using an AHC analysis, 3 groups (or clusters) were identified (Figure S3). Group 1 was composed of the unspoiled control samples which were located in the third quadrant. The PCA loading plot (Figure 5b) showed that the metabolites mainly associated with this group were lactic acid and 3,4-dimethylbenzaldehyde. Group 2 was composed of the samples GFL1, GFL2, MFL1, MFL2, GFC1, and GFC2, which were located in the second quadrant, and were mainly associated with the linear C_4_-C_7_ fatty acids and linear C_4_-C_6_ alcohols. Among these metabolites, butyric and valeric acids were predominant. Their concentrations in the samples of this group ranged from 440–770 mg L^−1^ (butyric acid) and from 792–1560 mg L^−1^ (valeric acid), which were much greater than their corresponding odor threshold in water (butyric acid, 1.40 mg L^−1^; valeric acid, 0.28 mg L^−1^) [34]. Therefore, it seems likely that these acids would have a noticeable impact on the olive odor. Caproic and heptanoic acids could also contribute to the odor as their concentrations were above the reported thresholds in water (caproic acid, 1.8 mg L^−1^; heptanoic acid, 0.64 mg L^−1^) [34,35]. Group 3 was composed of the rest of the spoiled samples, which were all located in the positive part of PC1. Two subgroups can be clearly observed within this group. One of them (GCC1, GCC2, GCL1, GCL2, MCL1, MCL2) was located in the positive part of PC2 and was characterized by high levels of cyclohexanoic acid (281–841 mg L^−1^), which was highly correlated to methyl cyclohexanecarboxylate, ethyl cyclohexanecarboxylate, and methyl hydrocinnamate. As mentioned in the Introduction section, cyclohexanoic acid is usually chosen as the reference for the “zapatera” off-odor descriptor in the sensory assessment of table olives. Although the odor threshold of this acid was not available, it can be inferred that such a threshold would be higher than 1.9 mg L^−1^ (concentration found in control M1, Table 3) but lower than 19.2 mg L^−1^ (=0.15 mM cyclohexanoic acid, reference concentration used for anchor point of 4). The second subgroup, which was located in the negative part of PC2, was composed of samples MCC1, MCC2, MFC1, and MFC2, and was mainly related to propyl acetate, (*Z*)-3-hexen-1-ol, and propyl propanoate. It is worth noting that these samples, except MCC1, had notable levels of cyclohexanoic acid (particularly sample MFC2 with 508 mg L^−1^) (Table 3).

### 2.4. Evaluation of Sensory Data

Based on the above-mentioned PCA results, 2 or 3 representatives of each group/subgroup were selected for sensory evaluation using the descriptors “zapatera”, “butyric”, and “putrid”. The mean scores for these spoilage descriptors are shown in Table 4. 

All samples had 1 or 2 spoilage descriptors with scores higher than the control sample. For the zapatera descriptor, all the samples except MFL1 and MCL2 had higher scores in comparison with the control. Samples GFC1, MFL1, and MCL2 had higher scores than the control for the butyric descriptor. However, the putrid perception was not noted in any of the samples. Therefore, only the sensory descriptors “butyric” and “zapatera” appeared to be adequate to characterize the spoiled samples. 

**Figure 6:**
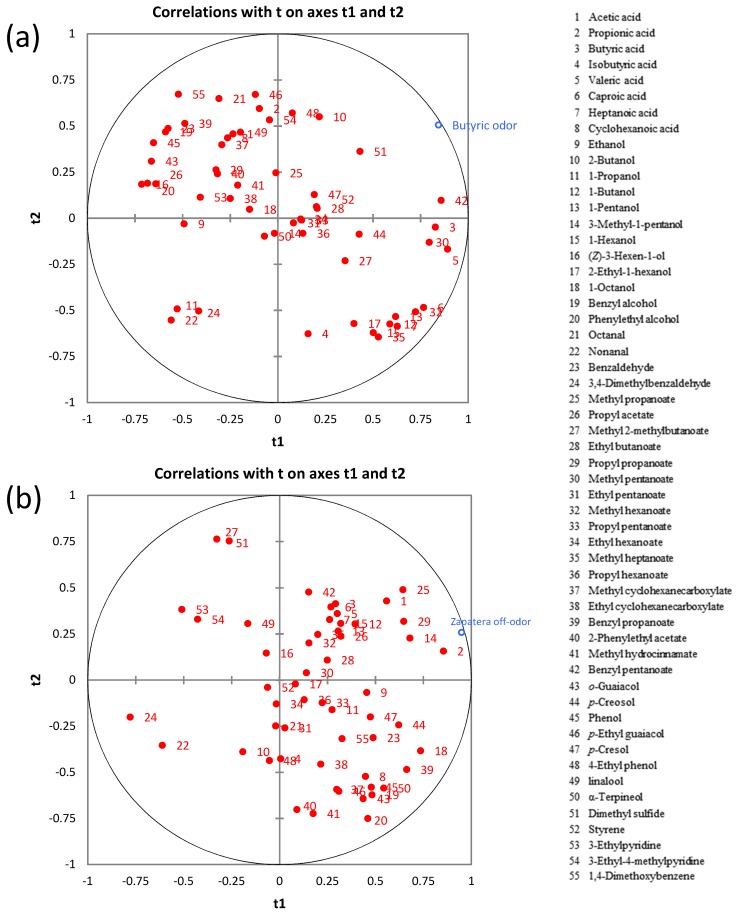
The PLS regression between the volatile metabolites and sensory spoilage descriptors: (**a**) butyric off-odor and (**b**) zapatera off-odor.

In order to reveal relationships between these descriptors (Y variables) and volatile metabolites (X variables), individual PLS analyses were carried out (Figure 6). 

The VIP (variable importance on projection) plots showing the important and significant X variables for butyric and zapatera descriptors are presented in Figure S4. Variables with VIP >1 are the most relevant for explaining the sensory spoilage descriptors. The main contributors to the butyric off-odor model were benzyl pentanoate, butyric acid, nonanal, valeric acid, 1-propanol, dimethyl sulfide, methyl pentanoate, (*Z*)-3-hexen-1-ol, phenylethyl alcohol, methyl hexanoate, and octanal. It is worth noting that a significant correlation between the butyric descriptor and butyric acid was found. This is not surprising, as this acid is the “character impact compound” of butyric spoilage. However, it must be pointed out that the number of samples used in the PLS analysis was small and when this fact occurs often makes it difficult to associate a single compound with a single sensory attribute, even if a relationship is known [36]. In the case of the zapatera descriptor, propionic acid, methyl propanoate, propyl propanoate, acetic acid, 3-methyl-1-pentanol, 1-octanol, α-terpineol, 1-butanol, and cyclohexanoic acid were the main contributors to the zapatera model. Cyclohexanoic acid, which is used as the reference material for the zapatera descriptor, as mentioned above, is included among the important contributors to the zapatera off-odor.

### 2.5. Correlation between Microbial Communities and Sensory Spoilage Descriptors

Pearson´s correlation coefficients between the mean values of the relative abundance of microorganisms and the intensity ratings of sensory spoilage descriptors are shown in Table S3. Considering only statistically significant correlations, it can be seen that the zapatera descriptor was positively correlated with *Propionibacterium* (r = 0.750, *p* < 0.05) and negatively correlated with *Natronobacillus* (r = −0.839, *p* < 0.01), *Oceanobacillus* (r = −0.957, *p* < 0.001), unclassified *Bacillaceae* (r = −0.874, *p* < 0.01), and *Candida etchellsii* (r = −0.767, *p* < 0.05). The positive correlation with *Propionibacterium* is not surprising, as zapatera spoilage is known to be caused by the participation of species of at least two genera of bacteria, *Clostridium* and *Propionibacterium* [37]. The butyric descriptor was positively correlated with *Ruminococcus* (r = 0.734, *p* < 0.05) and negatively correlated with *Lactobacillus* (r = −0.677, *p* < 0.05). *Ruminococcus* is a genus of bacteria within the order called Clostridiales. *Clostridium butyricum* and other species of *Clostridium* have been reported as microorganisms which are responsible for butyric spoilage in olives [37].

### 2.6. Correlation between Microbial Communities and Metabolites

**Figure 7:**
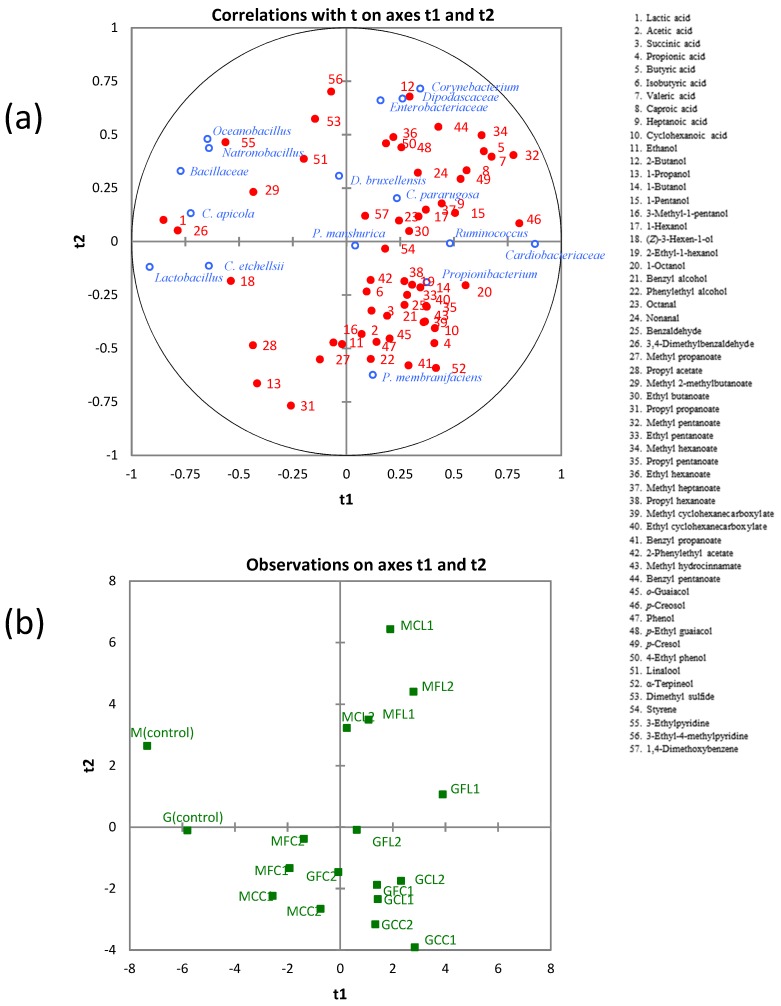
The PLS regression between microbial communities and metabolites. (**a**) Loading plot. (**b**) Score plot. See Table 5 for the meanings of samples abbreviations.

To reveal the relationships between microbial communities and metabolites, a PLS regression analysis was applied using the whole data set. Regarding bacterial communities, *Corynebacterium*, *Enterobacteriaceae*, *Ruminococcus*, *Cardiobacteriaceae*, and *Propionibacterium* were located in the right part; whereas *Natronobacillus*, *Oceanobacillus*, unclassified *Bacillaceae,* and *Lactobacillus* were in the left part of the correlation loading plot on the first two components (Figure 7). The latter group was positively correlated with lactic acid and 3,4-dimethylbenzaldehyde, which were highly correlated among themselves, as mentioned above. Furthermore, Pearson´s correlation coefficients between bacterial communities and metabolites (Table S4) showed that *Natronobacillus* and *Oceanobacillus* were positively correlated with 3-ethylpyridine; and *Lactobacillus* was positively correlated with (*Z*)-3-hexen-1-ol, propyl acetate, and 3-ethylpyridine. On the other hand, *Ruminococcus*, *Cardiobacteriaceae*, and *Propionibacterium* were negatively correlated with lactic acid. *Ruminococcus* did not show any significant positive correlation, although correlations for propionic acid (r = 0.462, *p* = 0.053) and butyric acid (r = 0.452, *p* = 0.059) were just over the limits of statistical significance.

*Cardiobacteriaceae* was positively correlated with butyric acid, valeric acid, caproic acid, 1-octanol, methyl pentanoate, methyl hexanoate, *p*-creosol, and *p*-cresol. *Propionibacterium* was positively correlated with acetic acid, propionic acid, succinic acid, and methyl propanoate. It is known that Propionibacteria are the microorganisms responsible for the development of the so-called “fourth stage” of fermentation in Spanish-style green table olives, with the formation of acetic and propionic acids occurring at the expense of lactic acid [38]. The genus *Corynebacterium* and family *Enterobacteriaceae* were negatively correlated with 1-propanol and propyl propanoate (Table S4). The genus *Corynebacterium* was positively correlated with butyric acid, 2-butanol, benzyl pentanoate, *p*-cresol, linalool, and 3-ethyl-4-methylpyridine. The family *Enterobacteriaceae* was positively correlated with 2-butanol, ethyl hexanoate, *p*-ethyl guaiacol, 4-ethyl phenol, 3-ethylpyridine, 3-ethyl-4-methylpyridine, and 1,4-dimethoxybenzene. 

Pearson’s correlation coefficients between yeast communities and metabolites are shown in Table S5. *Candida apicola* and *Candida etchellsii* were positively correlated with lactic acid and 3,4-dimethylbenzaldehyde. *Candida pararugosa* was positively correlated with methyl heptanoate. *Dekkera bruxellensis* and *Pichia manshurica* did not show any significant positive correlation. The family *Dipodascaceae* was positively correlated with butanoic acid, nonanal, *p*-cresol, and dimethyl sulfide. *Pichia membranifaciens* was positively correlated with α-terpineol.

## 3. Materials and Methods

### 3.1. Induction of Spoilage Fermentations

Manzanilla (M) and Gordal (G) cultivars, which are the most popular olive cultivars dedicated to the Spanish-style table olive processing in Spain, were used in the present study. Fruits were obtained from local growers in the Seville province during the ripening period, at the green stage and when they had reached normal size. After washing with tap water to eliminate plant materials and superficial contaminants, the olives from each cultivar were placed in vessels (3.3 kg of olives and 2.1 L of brine each) and treated in different ways in order to get different degrees or types of spoilage (Table 5). 

All treatments were carried out in duplicate. All samples were brined using a low initial brine concentration (71 g L^−1^ NaCl) to favor spoilage, except two replicate vessels from each cultivar which were covered with the habitual initial brine (120 g L^−1^ NaCl) and served as controls. These initial brine concentrations decreased when the balance between the surrounding brine and the olive flesh was reached (ca. 48 h), and fermentation and preservation took place at 35–39 g L^−1^ NaCl in all brines except the control brines, which contained 55–70 g L^−1^ NaCl during the first 8 months of fermentation. All the vessels were subjected to spontaneous fermentation without any starter application and left at room temperature for nearly one year. No correction was carried out except that the salt concentration and pH in the controls were adjusted to approximately 90 g L^−1^ and 3.9, respectively, by adding solid NaCl and a lactic acid solution after 8 months of brining. This is a recommended practice within the GMP with the aim of preventing spoilage throughout the summertime. Analyses of physicochemical and microbiological characteristics of the brines were carried out during the fermentation step (from the beginning to up to 7 months of brining). Analyses of metabolites and microbial DNA extraction from brines were carried out at the end of the post-fermentation step (11 months of brining), once the spoilages were clearly detected based on the detection of high pH values (above 4.5) and unpleasant (cheesy, zapatera) odors.

### 3.2. Chemical Analyses

The pH, titratable acidity, combined acidity, sodium chloride, and total polyphenols were measured following the routine procedures used in our laboratories [10]. Lactic acid, succinic acid, and ethanol were analyzed by HPLC using a C18 column and deionized water (pH adjusted to 2.2 using concentrated H_3_PO_4_) as the mobile phase and a refractive index detector [39].

Volatile compounds, except volatile fatty acids, were analyzed by HS-SPME-GC-MS following the procedure described in a previous work [14] with modifications. Brine samples (≈50 mL) were neutralized to pH 7–8 with magnesium oxide and the aid of a pH meter and magnetic stirrer. The precipitate was separated by filtration and an aliquot of filtrate (2.5 mL) was inserted into a 15 mL glass vial. After the addition of 7.5 mL of a NaCl solution (300 g L^−1^) and 100 µL of 3-octanol (2 mg L^−1^), the vial was closed and placed in a water bath adjusted to 40 °C. The vial was equilibrated for 15 min at 40 °C and stirred at 600 rpm using a stirring bar. The headspace volatile compounds were extracted for 30 min on a divinylbenzene/carboxen/polydimethylsiloxane (DVB/CAR/PDMS) fiber (1 cm, 50/30 µm; Supelco, Bellefonte, PA, USA). The volatile compounds adsorbed on the SPME fiber were desorbed at 265 °C for 15 min in the injector port of a GC interfaced with a mass detector (internal ionization source: 70 eV) with a scan range from *m*/*z* 30 to 400 (GC model 7890A and mass detector model 5975C, Agilent Technologies, Santa Clara, CA, USA). The chromatographic conditions along with the identification and quantification procedures are described in [14]. Three replicates per each sample were prepared and analyzed.

Volatile fatty acids were analyzed by HS-SPME-GC-MS using a new procedure. An aliquot (2.5 mL) of brine, 0.2 mL of 2 N HCl, and 7.3 mL of a NaCl solution (300 g L^−1^) were placed in a 15 mL vial. As the internal standard, 20 µL of 2-ethylbutyric acid (900 mg L^−1^) were used. After equilibration for 15 min at 40 °C, a polyacrylate-coated SPME fiber (PA, 85 µm; Supelco) was exposed to the sample HS for 30 min at 40 °C. Volatile acids were desorbed at the GC injection port at 250 °C for 15 min. The GC-MS system was equipped with a DB-FFAP column (30 m × 0.25 mm i.d., 0.25 µm; J&W Scientific, Folsom, CA, USA). The helium flow rate was 1 mL/min. The column was maintained at 80 °C for 2 min, ramped at 8 °C/min to 200 °C, and held for 15 min. The transfer line temperature was maintained at 230 °C. For the mass detector conditions, the quadrupole and ion source temperatures were maintained at 150 and 230 °C, respectively. The concentrations of the volatile fatty acids were calculated from calibration curves with standards of acetic, propionic, isobutyric, butyric, 3-methylbutyric, 2-ethylbutyric (IS), valeric, 4-methylvaleric, caproic, heptanoic, and cyclohexanoic acids and expressed in mg L^−1^. The following equation was used:

$${\left[x\right]}_{\mathrm{sample}}=\mathrm{peak}\;\mathrm{area}\;\left(x\right)\times \frac{\left[\mathrm{IS}\right]}{\mathrm{peak}\;\mathrm{area}\;\left(\mathrm{IS}\right)}\times \frac{\mathrm{slope}\;\left(\mathrm{IS}\right)}{\mathrm{slope}\;\left(x\right)}$$

where [x]_sample_ is the concentration of the compound in the brine sample, [IS] is the concentration of the internal standard (IS), and slope (IS) and slope (x) are slopes from the calibration curves of the internal standard and compound x, respectively. The calibration curves gave coefficients of determination (*R*^2^) ranging from 0.988 to 0.999 and detection limits from 0.02 to 7 mg L^−1^. The relative standard deviation (RSD) ranged from 2 to 11%, and recovery rates from 91 to 111%. Each sample was analyzed in triplicate. Spoilage samples were also analyzed at a 1:50 dilution to account for acids present in amounts that resulted in the column overloading in the undiluted sample.

### 3.3. Microbiological Analyses

Both culture-dependent and -independent techniques were applied to investigate the microbiota present in the different vessels. The viable and culturable populations of lactic acid bacteria (LAB), and yeast and molds were determined by plating the brines and their decimal dilutions (in 0.9% NaCl) with a Spiral Plater (Don Whitley Sci. Ltd., Shipley, UK). The culture media used were De Man, Rogosa, Sharpe (MRS) agar (Biokar diagnostics, Beauvais, France) with and without 0.02% sodium azide (Sigma-Aldrich) and oxytetracycline-glucose-yeast extract (Oxoid Ltd., Basingstoke, UK) agar for the aforementioned groups, respectively. MRS plates were incubated under anaerobic conditions (AnaeroGen, Oxoid) and OGYE plates in aerobiosis, all at 32 °C for up to five days, and the numbers of colony forming units were counted with a Scan 500 (Interscience, St Nom la Bretèche, France) colony counter.

Microbial DNA extraction, preparation of libraries and the MiSeq Sequencing of brine samples collected from the different vessels were used for bacterial and fungal community analyses. DNA extraction was performed as described in Reference [40]. Ten mL of brine were spun at 9000× *g* for 20 min at 5 °C. Then the pellet was washed twice in saline solution (9 g L^−1^ NaCl). DNA isolation was done using the PowerFood^®^ Microbial DNA Isolation Kit (MoBio, Carlsbad, CA, USA) according to the manufacturer’s instructions. Purified DNA samples were sent to the Sequencing and Bioinformatics Service of FISABIO (Valencia, Spain) for the massive sequencing of 16S rDNA (the gene that codes for the 16S ribosomal RNA) and ITS (Internal Transcribed Spacer) amplicons using a MiSeq Illumina platform. Metagenomic libraries and sequencing were performed as described in Reference [29]. The bioinformatic analysis was also carried out at FISABIO using an ad-hoc pipeline written in the R statistics language [41]. The taxonomic affiliations of 16S rDNA and ITS datasets were assigned using the RDP (Recombination Detection Program) classifier from the Ribosomal Database [42,43] and the UNITE fungal classification database (https://unite.ut.ee/), respectively.

### 3.4. Sensory Analysis

The selected brine samples were subjected to a sensory evaluation of odor by a trained panel composed of 15 judges (9 males and 6 females). The judges were familiarized with the descriptive analysis technique. Three off-odor descriptors (“zapatera”, “butyric”, and “putrid”) were evaluated by the panel. The references for each descriptor were those used in the guidelines for the taster and panel leader training in the sensory assessment of table olives and panel management according to the standard COI/OT/MO Doc. No 1-2011 [6]. For zapatera spoilage, two aqueous solutions of cyclohexanoic acid at 0.15 and 1.0 mM for anchor points of 4 and 9, respectively, in a linear scale from 1 to 11, were used as references. For butyric spoilage, 0.5 and 1.0 mM solutions of butyric acid for anchor points of 3.5 and 7.0, respectively, were used as references. For putrid spoilage, 0.1 and 1.0 mM solutions of 2-mercaptoethanol for anchor points of 2.5 and 8.0, respectively, were used as references. The brines were presented in cups coded with a 3-digit random number and covered with a lid in individual booths under incandescent white light at the sensory laboratory of our department at the Instituto de la Grasa. The judges were asked to score the brines according to a 10-cm unstructured scale. Anchor ratings were 1 (no perception) and 11 (extremely strong). The marks in the questionnaire were transformed into data by taking measurements (in 0.1 cm) from the left anchor. The evaluations were performed in duplicate. Mean scores (panel average) for each descriptor were obtained and used for further analysis.

### 3.5. Statistical Analyses

Principal component analysis (PCA) was performed to assess the internal degree of correlation between the variables in the chemical and sensory data. Agglomerative hierarchical cluster (AHC) analysis, using Euclidian distance and Ward´s method as a similarity criterion, was carried out to cluster the samples in homogenous groups. The partial least square (PLS) regression technique and Pearson´s correlation test were used to find correlations among metabolites, sensory descriptors, and microbial communities. All the above-mentioned statistical analyses were performed with XLSTAT v. 2016 (Addinsoft, New York, NY, USA). The Dunnett´s test was used to compare the mean scores of sensory spoilage descriptors from selected samples against an unspoiled control. This test was performed with SPSS software v. 23.0 (IBM Corp., Armonk, NY, USA). Significant differences were determined at the *p* < 0.05 level.

## 4. Conclusions

The microbiota and metabolites in spoiled Spanish-style table olives were disclosed, along with their relationships in the sensory evaluation and the correlations among them. A low salt concentration resulted in the main factor for prompting spoilage, whereas different alkaline and washing steps, which are crucial for this type of elaboration, were not significant in preventing or favoring deterioration. Some unexpected bacterial taxa, such as *Cardiobacteriaceae* and *Ruminococcus* turned out to be the most abundant microorganisms once the spoilage took place, and coexisted with *Pichia membranifaciens*, a common yeast species in table olives. The disappearance of lactic acid in the spoiled samples was the foremost metabolite difference between the spoiled and control samples. In addition, all the spoiled samples had higher contents of volatile acids and phenols than the control samples. Several volatile acids could contribute to the olive’s off-odor as their concentrations were above the reported thresholds in water. Although the number of samples used in the present study was small, the associations found among microbial communities, metabolites, and sensory spoilage descriptors appeared quite reasonable in light of previous studies. Thus, it is not surprising that butyric and cyclohexanoic acids were among the main contributors to the butyric and zapatera off-odors, respectively. The butyric attribute exhibited a significant positive relationship with the genus *Ruminococcus*; whereas the genus *Propionibacterium* was positively correlated with the zapatera descriptor. In addition, the statistical analyses revealed that the *Propionibacterium* was positively correlated with acetic acid, propionic acid, succinic acid, and methyl propanoate. The propionic and butyric acids appeared to be correlated with *Ruminococcus*, although the correlations were at the margin of significance. On the other hand, *Cardiobacteriaceae* was positively correlated with butyric acid, valeric acid, caproic acid, 1-octanol, methyl pentanoate, methyl hexanoate, *p*-creosol, and *p*-cresol. Some of these compounds could be metabolic end-products generated as a result of the lactic acid metabolism by these bacteria. However, further studies concerning bacterial metabolism are required to confirm this hypothesis.

## Figures and Tables

**Table 1:** The physicochemical analysis of olive brines after 11 months of brining.

Sample ^a^	pH	Titratable Acidity (g L^−1^ Lactic Acid)	Combined Acidity (eq L^−1^)	Salt (g L^−1^ NaCl)	Total Phenols (g L^−1^ Gallic Acid)
**Gordal Cultivar**					
G1 (control)	4.53	3.6	0.082	87	1.39
G2 (control)	4.67	3.6	0.080	88	1.39
GFC1	4.99	4.3	0.130	38	1.72
GFC2	5.05	4.4	0.127	37	2.17
GFL1	5.10	2.4	0.086	37	1.57
GFL2	4.86	4.5	0.097	37	1.50
GCC1	5.06	3.9	0.121	38	2.48
GCC2	4.95	4.8	0.119	38	2.54
GCL1	4.83	3.8	0.076	35	1.74
GCL2	4.84	4.0	0.081	39	1.64
**Manzanilla Cultivar**					
M1 (control)	4.27	4.5	0.081	87	2.00
M2 (control)	4.19	4.5	0.078	87	2.05
MFC1	4.84	6.5	0.134	36	3.59
MFC2	4.92	5.2	0.128	37	3.34
MFL1	4.96	3.7	0.134	36	2.07
MFL2	5.01	3.4	0.128	37	2.25
MCC1	4.66	5.9	0.113	39	3.90
MCC2	4.73	6.1	0.108	38	3.78
MCL1	4.96	3.0	0.085	35	2.90
MCL2	4.89	3.6	0.083	37	2.91

^a^ See Table 5 for the meanings of samples abbreviations.

**Table 2:** The concentration of metabolites in spoiled and unspoiled (control) brine samples from the Gordal cultivar.

Compounds	Samples ^a^									
	G1 (control)	G2 (control)	GFC1	GFC2	GFL1	GFL2	GCC1	GCC2	GCL1	GCL2
**Non-volatile acids ^b^**										
Lactic acid	5.20 ± 0.20	3.70 ± 0.08	ND	ND	ND	ND	ND	ND	ND	ND
Succinic acid	0.24 ± 0.09	0.16 ± 0.01	0.33 ± 0.03	0.27 ± 0.01	0.27 ± 0.01	0.22 ± 0.01	0.22 ± 0.01	0.23 ± 0.00	0.12 ± 0.00	0.12 ± 0.00
**Volatile acids ^c^**										
Acetic acid	891 ± 32	1244 ± 83	4558 ± 880	3528 ± 182	1392 ± 164	2322 ± 511	2161 ± 415	2446 ± 19	1782 ± 274	3107 ± 536
Propionic acid	1077 ± 22	1854 ± 125	5378 ± 850	5284 ± 240	3022 ± 98	3727 ± 391	4216 ± 109	4302 ± 192	2846 ± 127	3795 ± 115
Isobutyric acid	1.6 ± 0.3	3.1 ± 0.1	ND	5 ± 2	3.3 ± 0.3	ND	3.5 ± 0.0	2.3 ± 0.0	ND	1.9 ± 0.8
Butyric acid	6.6 ± 0.5	12 ± 4	603 ± 80	440 ± 12	630 ± 49	538 ± 16	185 ± 22	135 ± 1	164 ± 10	313 ± 3
Valeric acid	0.9 ± 0.5	1 ± 1	1068 ± 127	792 ± 73	1560 ± 26	1001 ± 62	386 ± 39	256 ± 2	350 ± 7	513 ± 50
Caproic acid	0.2 ± 0.0	0.2 ± 0.1	65 ± 7	38 ± 5	194 ± 6	59 ± 1	10 ± 1	6 ± 2	11.4 ± 0.4	25 ± 2
Heptanoic acid	ND	ND	2.2 ± 0.4	1.3 ± 0.3	11.1 ± 0.1	1.1 ± 0.2	0.2 ± 0.0	0.1 ± 0.0	0.2 ± 0.0	0.3 ± 0.0
Cyclohexanoic acid	1.0 ± 0.5	0.6 ± 0.5	422 ± 16	31 ± 10	61 ± 9	0.6 ± 0.8	841 ± 100	729 ± 31	401 ± 1	479 ± 56
**Alcohols ^d^**										
Ethanol ^b^	0.12 ± 0.02	0.14 ± 0.03	0.44 ± 0.02	0.27 ± 0.07	0.15 ± 0.01	0.23 ± 0.05	0.28 ± 0.02	0.27 ± 0.03	0.46 ± 0.06	0.45 ± 0.01
2-Butanol	8 ± 1	6.5 ± 0.2	14 ± 2	10.2 ± 0.1	14 ± 3	26.4 ± 0.3	55 ± 5	16.8 ± 0.8	26 ± 4	36.5 ± 0.8
1-Propanol	46 ± 2	55.8 ± 0.5	42 ± 5	42.7 ± 0.2	31 ± 6	45 ± 1	43 ± 3	19 ± 2	36 ± 5	29.5 ± 0.5
1-Butanol	1 ± 1	2.44 ± 0.01	3.83 ± 0.06	5.3 ± 0.2	9.6 ± 2	4.30 ± 0.05	2 ± 1	0.85 ± 0.04	4 ± 1	1.69 ± 0.07
1-Pentanol	ND	ND	7.9 ± 0.5	17.5 ± 0.3	38 ± 8	8.69 ± 0.08	6 ± 3	2.15 ± 0.09	2.4 ± 0.4	2.79 ± 0.06
3-Methyl-1-pentanol	0.3 ± 0.4	0.1 ± 0.1	0.62 ± 0.02	0.52 ± 0.07	0.37 ± 0.05	0.38 ± 0.01	0.2 ± 0.2	0.29 ± 0.08	0.32 ± 0.07	0.13 ± 0.06
1-Hexanol	0.90 ± 0.09	0.91 ± 0.03	1.42 ± 0.03	1.58 ± 0.04	5.1 ± 0.7	1.40 ± 0.01	1.2 ± 0.6	0.5 ± 0.2	0.92 ± 0.09	0.80 ± 0.03
(*Z*)-3-Hexen-1-ol	3.00 ± 0.02	2.7 ± 0.1	3.8 ± 0.3	3.6 ± 0.2	2.1 ± 0.3	2.7 ± 0.1	4 ± 2	3.5 ± 0.2	3.7 ± 0.1	2.6 ± 0.1
2-Ethyl-1-hexanol	5.8 ± 0.2	5.9 ± 0.3	3.40 ± 0.08	4.32 ± 0.05	15.5 ± 0.3	17.4 ± 0.1	4 ± 1	2.4 ± 0.1	28 ± 2	17.5 ± 0.3
1-Octanol	0.53 ± 0.00	0.55 ± 0.01	0.81 ± 0.03	0.69 ± 0.02	0.86 ± 0.02	0.68 ± 0.04	1.0 ± 0.2	0.78 ± 0.02	1.1 ± 0.5	0.62 ± 0.06
Benzyl alcohol	4.41 ± 0.08	4.3 ± 0.3	7.6 ± 0.6	3.8 ± 0.1	4.4 ± 0.9	6.0 ± 0.1	14 ± 6	8.8 ± 0.5	9.3 ± 0.6	6.1 ± 0.1
Phenylethyl alcohol	7.97 ± 0.07	8.1 ± 0.5	12.8 ± 0.9	11.2 ± 0.3	7 ± 1	6.9 ± 0.2	33 ± 14	20 ± 1	15 ± 1	11.0 ± 0.2
**Aldehydes ^d^**										
Octanal	0.2 ± 0.2	0.21 ± 0.08	0.31 ± 0.04	0.3 ± 0.1	ND	0.3 ± 0.2	0.50 ± 0.02	0.51 ± 0.04	1.2 ± 0.5	0.4 ± 0.2
Nonanal	0.66 ± 0.06	0.68 ± 0.05	1.0 ± 0.2	1.13 ± 0.06	1.3 ± 0.5	0.9 ± 0.1	1.58 ± 0.08	1.6 ± 0.1	1.7 ± 0.4	1.34 ± 0.01
Benzaldehyde	7.6 ± 0.2	3.5 ± 0.2	7.2 ± 0.7	7.8 ± 0.5	1.8 ± 0.5	14.6 ± 0.1	17 ± 8	12.5 ± 0.3	16.4 ± 0.4	6 ± 1
3,4-Dimethylbenzaldehyde	1.7 ± 0.2	1.49 ± 0.04	ND	ND	ND	ND	0.44 ± 0.06	ND	0.7 ± 0.6	0.15 ± 0.02
**Esters ^d^**										
Methyl propanoate	28 ± 2	28.26 ± 0.01	47.7 ± 0.3	33.6 ± 0.7	32 ± 7	19 ± 5	22 ± 4	43 ± 0.9	26 ± 5	15.9 ± 0.6
Propyl acetate	24 ± 3	19.9 ± 0.6	20 ± 1	12.35 ± 0.08	5 ± 1	14.4 ± 0.2	15 ± 1	8.3 ± 0.4	15 ± 2	9.7 ± 0.2
Methyl 2-methylbutanoate	3.2 ± 0.4	3.15 ± 0.03	2.5 ± 0.1	3.2 ± 0.2	3.6 ± 0.3	3.0 ± 0.1	1.1 ± 0.1	2.2 ± 0.1	2.5 ± 0.3	1.51 ± 0.07
Ethyl butanoate	ND	ND	10.27 ± 0.03	ND	ND	ND	ND	ND	ND	15.2 ± 0.2
Propyl propanoate	82 ± 5	88 ± 1	110 ± 4	67.20 ± 0.01	28 ± 2	63.4 ± 0.6	44 ± 5	58 ± 1	68 ± 6	62.8 ± 0.8
Methyl pentanoate	0.75 ± 0.00	0.53 ± 0.03	25 ± 5	21 ± 6	66 ± 5	31 ± 3	23 ± 3	36.0 ± 0.2	58 ± 2	70 ± 2
Ethyl pentanoate	0.4 ± 0.6	0.64 ± 0.09	1.6 ± 0.4	ND	ND	ND	10 ± 5	9.8 ± 0.2	37 ± 1	81 ± 6
Methyl hexanoate	ND	ND	8.3 ± 0.7	9 ± 2	88 ± 6	19 ± 2	6 ± 2	7.7 ± 0.1	19 ± 2	56.6 ± 0.4
Propyl pentanoate	0.3 ± 0.4	0.58 ± 0.01	74 ± 14	18 ± 3	9.0 ± 0.2	48.9 ± 0.9	36 ± 16	26.1 ± 0.4	84 ± 10	201 ± 14
Ethyl hexanoate	ND	ND	ND	ND	ND	ND	ND	ND	ND	4.5 ± 0.8
Methyl heptanoate	ND	ND	ND	ND	2.4 ± 0.3	0.2 ± 0.2	ND	ND	ND	ND
Propyl hexanoate	ND	ND	1.94 ± 0.09	0.2 ± 0.3	0.39 ± 0.09	1.3 ± 0.1	0.7 ± 0.4	0.42 ± 0.06	2.1 ± 0.5	10.0 ± 0.2
Methyl cyclohexanecarboxylate	0.43 ± 0.02	0.39 ± 0.02	13 ± 2	0.9 ± 0.2	2.3 ± 0.1	0.04 ± 0.01	54 ± 3	83.9 ± 0.4	48 ± 2	45.7 ± 0.7
Ethyl cyclohexanecarboxylate	0.34 ± 0.01	0.27 ± 0.01	1.11 ± 0.01	ND	ND	ND	26 ± 9	31.6 ± 0.4	33 ± 1	47 ± 4
Benzyl propanoate	ND	ND	0.82 ± 0.00	0.40 ± 0.03	0.36 ± 0.05	0.43 ± 0.01	1.9 ± 0.4	1.8 ± 0.1	0.98 ± 0.09	0.77 ± 0.04
2-Phenylethyl acetate	1.23 ± 0.03	1.22 ± 0.04	1.97 ± 0.00	1.6 ± 0.2	0.5 ± 0.3	0.96 ± 0.08	6 ± 2	4.6 ± 0.3	3.53 ± 0.02	1.22 ± 0.02
Methyl hydrocinnamate	ND	ND	0.10 ± 0.04	0.05 ± 0.01	0.12 ± 0.03	0.09 ± 0.01	1.2 ± 0.5	1.09 ± 0.06	1.32 ± 0.08	1.54 ± 0.09
Benzyl pentanoate	ND	ND	0.62 ± 0.09	ND	0.55 ± 0.01	0.46 ± 0.01	ND	ND	ND	ND
**Phenols ^d^**										
*o*-Guaiacol	ND	ND	2.55 ± 0.03	0.94 ± 0.07	0.9 ± 0.2	3.07 ± 0.06	86 ± 4	69 ± 4	28 ± 3	11.1 ± 0.1
*p*-Creosol	70 ± 2	65 ± 4	134 ± 3	150 ± 7	196 ± 3	171 ± 3	185 ± 3	116 ± 6	124 ± 8	121.77 ± 0.03
Phenol	1.0 ± 0.1	0.95 ± 0.03	4.3 ± 0.2	1.21 ± 0.09	2.5 ± 0.4	2.98 ± 0.08	28 ± 11	18 ± 1	14 ± 1	10.1 ± 0.1
*p*-Ethyl guaiacol	0.15 ± 0.02	0.14 ± 0.03	0.35 ± 0.02	0.20 ± 0.09	0.15 ± 0.06	0.18 ± 0.07	0.6 ± 0.2	0.3 ± 0.1	0.51 ± 0.03	0.34 ± 0.00
*p*-Cresol	0.74 ± 0.00	0.71 ± 0.05	1.47 ± 0.05	1.92 ± 0.09	2.8 ± 0.5	1.8 ± 0.1	3 ± 1	1.81 ± 0.09	1.5 ± 0.1	1.51 ± 0.05
4-Ethyl phenol	1.09 ± 0.06	1.1 ± 0.1	8.5 ± 0.4	2.1 ± 0.2	1.8 ± 0.4	1.84 ± 0.04	16 ± 6	4.6 ± 0.3	6.9 ± 0.3	5.15 ± 0.03
**Terpenes ^d^**										
Linalool	ND	ND	0.38 ± 0.02	0.2 ± 0.1	0.33 ± 0.03	0.35 ± 0.01	ND	ND	ND	ND
α-Terpineol	4.17 ± 0.09	4.1 ± 0.1	7.4 ± 0.1	7.12 ± 0.05	5.7 ± 0.3	5.5 ± 0.1	14 ± 5	8.0 ± 0.4	6.39 ± 0.08	5.99 ± 0.05
**Others ^d^**										
Dimethyl sulfide	1.3 ± 0.2	1.17 ± 0.09	1.6 ± 0.1	1.11 ± 0.05	1.4 ± 0.2	1.74 ± 0.05	0.6 ± 0.4	1.06 ± 0.02	1.42 ± 0.08	1.50 ± 0.03
Styrene	4.3 ± 0.1	2.29 ± 0.03	2 ± 3	2.8 ± 0.3	2.3 ± 0.6	4.1 ± 0.8	1.1 ± 1.5	9.5 ± 0.2	3.8 ± 0.9	10.9 ± 0.3
3-Ethylpyridine	2.70 ± 0.02	2.2 ± 0.2	1.77 ± 0.03	1.5 ± 0.1	1.3 ± 0.2	0.84 ± 0.03	0.6 ± 0.9	0.84 ± 0.03	0.78 ± 0.01	1.01 ± 0.01
3-Ethyl-4-methylpyridine	1.79 ± 0.06	1.3 ± 0.1	4.0 ± 0.1	1.8 ± 0.2	1.6 ± 0.2	1.05 ± 0.03	1.4 ± 0.7	0.9 ± 0.1	0.84 ± 0.01	1.1 ± 0.1
1,4-Dimethoxybenzene	1.75 ± 0.07	1.90 ± 0.08	2 ± 0.2	2.1 ± 0.3	1.7 ± 0.1	2.13 ± 0.05	3 ± 1	2.1 ± 0.2	2.1 ± 0.1	1.96 ± 0.02

^a^ Values are means ± SD. ND = not detected. See Table 5 for the meanings of samples abbreviations. ^b^ Quantitative determination expressed in g L^−1^. ^c^ Quantitative determination expressed in mg L^−1^. ^d^ Semi-quantitative determination expressed as µg L^−1^ of 3-octanol.

**Table 3:** The concentration of metabolites in spoiled and unspoiled (control) brine samples from the Manzanilla cultivar.

	Samples ^a^									
Compounds	M1 (control)	M2 (control)	MFC1	MFC2	MFL1	MFL2	MCC1	MCC2	MCL1	MCL2
**Non-volatile acids ^b^**										
Lactic acid	7.11 ± 0.05	7.8 ± 0.2	ND	ND	ND	ND	3.9 ± 0.1	ND	ND	ND
Succinic acid	0.12 ± 0.01	0.09 ± 0.03	0.25 ± 0.01	0.21 ± 0.00	0.11 ± 0.00	0.09 ± 0.00	0.20 ± 0.01	0.31 ± 0.02	0.23 ± 0.01	0.25 ± 0.01
**Volatile acids ^c^**										
Acetic acid	1435 ± 372	882 ± 142	4809 ± 632	4411 ± 151	2589 ± 662	2762 ± 367	3206 ± 808	4236 ± 200	1510 ± 35	1367 ± 173
Propionic acid	858 ± 174	546 ± 78	5588 ± 236	5031 ± 54	3515 ± 647	3747 ± 55	2701 ± 284	5473 ± 603	2209 ± 4	3262 ± 635
Isobutyric acid	1 ± 1	ND	ND	ND	2.4 ± 0.8	1.5 ± 0.4	1.9 ± 2.6	ND	ND	ND
Butyric acid	2.8 ± 0.9	2.5 ± 0.3	12.4 ± 0.2	132 ± 2	769 ± 141	744 ± 6	13 ± 1	13.5 ± 0.9	402 ± 15	139 ± 4
Valeric acid	2 ± 1	0.5 ± 0.1	4.2 ± 0.2	177 ± 7	1215 ± 149	1407 ± 103	4 ± 3	1.1 ± 0.5	598 ± 10	482 ± 1
Caproic acid	0.2 ± 0.0	0.1 ± 0.0	0.3 ± 0.0	3.9 ± 0.4	76 ± 9	97.6 ± 0.3	0.8 ± 0.4	0.3 ± 0.1	32 ± 5	9 ± 2
Heptanoic acid	ND	ND	ND	ND	1.1 ± 0.1	2.7 ± 0.2	ND	ND	0.4 ± 0.0	0.2 ± 0.0
Cyclohexanoic acid	2 ± 1	ND	89 ± 1	508 ± 32	48 ± 8	2.6 ± 0.4	1.6 ± 0.2	72 ± 6	280 ± 37	370 ± 12
**Alcohols ^d^**										
Ethanol	0.22 ± 0.03	0.23 ± 0.05	0.12 ± 0.02	0.13 ± 0.01	0.07 ± 0.00	0.08 ± 0.01	0.95 ± 0.07	0.89 ± 0.05	0.34 ± 0.05	0.06 ± 0.02
2-Butanol	6 ± 2	12.3 ± 0.8	10.7 ± 0.5	14.4 ± 0.4	45 ± 13	83 ± 1	15 ± 1	20.9 ± 0.9	259 ± 13	190 ± 5
1-Propanol	42 ± 16	46 ± 7	20 ± 1	24.2 ± 0.6	3.0 ± 0.3	2.7 ± 0.1	49 ± 4	35 ± 1	ND	3.0 ± 0.2
1-Butanol	0.7 ± 0.3	0.5 ± 0.7	ND	1.05 ± 0.03	2.2 ± 0.4	ND	2.0 ± 0.3	1.0 ± 0.2	ND	ND
1-Pentanol	ND	ND	ND	4.44 ± 0.09	6 ± 1	11.16 ± 0.05	ND	ND	4 ± 2	3.1 ± 0.1
3-Methyl-1-pentanol	ND	ND	ND	ND	ND	0.2 ± 0.2	1.28 ± 0.02	0.64 ± 0.04	ND	ND
1-Hexanol	0.8 ± 0.1	1.48 ± 0.01	0.69 ± 0.02	1.16 ± 0.03	1.2 ± 0.1	1.87 ± 0.02	1.38 ± 0.06	1.2 ± 0.1	0.9 ± 0.3	0.47 ± 0.08
(*Z*)-3-Hexen-1-ol	8 ± 1	10.8 ± 0.7	9.2 ± 0.3	10.6 ± 0.2	3.2 ± 0.5	2 ± 2	11.1 ± 0.5	12 ± 1	6 ± 2	4.7 ± 0.3
2-Ethyl-1-hexanol	5.0 ± 0.5	5.32 ± 0.07	2.7 ± 0.2	2.42 ± 0.01	2.93 ± 0.09	2.8 ± 0.1	2.4 ± 0.2	3.9 ± 0.5	2.6 ± 0.4	5.2 ± 0.1
1-Octanol	0.5 ± 0.1	0.4 ± 0.2	0.6 ± 0.1	0.68 ± 0.06	0.66 ± 0.00	0.9 ± 0.4	1.00 ± 0.03	0.9 ± 0.1	0.8 ± 0.3	0.76 ± 0.05
Benzyl alcohol	5.0 ± 0.5	6.5 ± 0.1	11 ± 1	8.29 ± 0.07	7.7 ± 0.1	6.1 ± 0.5	8.5 ± 0.6	11 ± 1	8.8 ± 0.7	7.6 ± 0.3
Phenylethyl alcohol	10 ± 1	12.9 ± 0.2	14 ± 1	11.15 ± 0.02	9.0 ± 0.1	8.8 ± 0.4	20 ± 1	22 ± 4	14.2 ± 0.9	11.72 ± 0.06
**Aldehydes ^d^**										
Octanal	0.2 ± 0.1	0.50 ± 0.09	0.3 ± 0.1	0.4 ± 0.1	0.6 ± 0.1	1 ± 1	0.4 ± 0.1	0.41 ± 0.03	0.5 ± 0.4	0.37 ± 0.03
Nonanal	1.9 ± 0.8	0.97 ± 0.07	1.1 ± 0.3	1.3 ± 0.4	1.3 ± 0.1	4 ± 4	1.11 ± 0.02	1 ± 1	1.3 ± 0.1	1.11 ± 0.05
Benzaldehyde	2.5 ± 0.4	4 ± 2	14.9 ± 0.8	13.5 ± 0.8	11.7 ± 0.6	13 ± 2	3.2 ± 0.7	17 ± 3	9.1 ± 0.2	5.8 ± 0.4
3,4-Dimethylbenzaldehyde	1.8 ± 0.1	2.1 ± 0.3	0.61 ± 0.03	ND	0.3 ± 0.1	ND	0.19 ± 0.09	0.1 ± 0.1	ND	ND
**Esters ^d^**										
Methyl propanoate	14 ± 5	25 ± 2	55 ± 5	41 ± 1	12 ± 2	24 ± 1	32 ± 3	36.7 ± 0.4	10 ± 7	22 ± 1
Propyl acetate	15 ± 3	16 ± 1	30 ± 2	48 ± 1	2.0 ± 0.5	1.79 ± 0.08	73 ± 3	51 ± 2	1.2 ± 0.6	1.0 ± 0.1
Methyl 2-methylbutanoate	3.1 ± 0.7	3.6 ± 1.0	3.0 ± 0.5	3.1 ± 0.1	3.1 ± 0.4	3.36 ± 0.04	3.37 ± 0.08	2.8 ± 0.3	1.7 ± 0.7	2.6 ± 0.2
Ethyl butanoate	ND	ND	ND	ND	ND	ND	ND	ND	9 ± 3	ND
Propyl propanoate	16 ± 3	13 ± 1	98 ± 8	94 ± 2	4.9 ± 0.7	4.79 ± 0.04	102 ± 4	98 ± 10	4 ± 1	8.34 ± 0.08
Methyl pentanoate	ND	ND	0.56 ± 0.02	12.3 ± 0.1	52 ± 4	66 ± 1	0.41 ± 0.02	0.1 ± 0.1	59 ± 19	42.3 ± 0.1
Ethyl pentanoate	ND	ND	0.9 ± 0.1	ND	3.6 ± 0.1	0.34 ± 0.01	0.52 ± 0.01	0.1 ± 0.1	ND	12.4 ± 0.1
Methyl hexanoate	ND	ND	ND	3.39 ± 0.01	40 ± 5	42.1 ± 0.2	1.35 ± 0.05	0.59 ± 0.03	65 ± 9	11.1 ± 0.4
Propyl pentanoate	ND	ND	0.3 ± 0.4	7.0 ± 0.1	7.4 ± 0.4	3.9 ± 0.4	0.2 ± 0.2	ND	17 ± 2	7.6 ± 0.2
Ethyl hexanoate	ND	ND	ND	0.37 ± 0.00	0.24 ± 0.00	ND	0.41 ± 0.03	ND	11.7 ± 0.9	0.69 ± 0.08
Methyl heptanoate	ND	ND	ND	ND	ND	0.4 ± 0.5	ND	ND	ND	ND
Propyl hexanoate	ND	ND	ND	ND	0.49 ± 0.00	ND	ND	ND	0.8 ± 0.1	ND
Methyl cyclohexanecarboxylate	0.21 ± 0.06	0.10 ± 0.00	11.9 ± 0.4	37 ± 2	3.51 ± 0.01	0.1 ± 0.1	0.2 ± 0.2	8.0 ± 0.7	26 ± 1	31 ± 1
Ethyl cyclohexanecarboxylate	0.19 ± 0.04	ND	5.2 ± 0.1	18.2 ± 0.1	0.29 ± 0.03	ND	0.2 ± 0.2	6.1 ± 0.4	22.3 ± 0.7	8.3 ± 0.7
Benzyl propanoate	ND	ND	1.6 ± 0.1	1.29 ± 0.01	0.57 ± 0.01	0.25 ± 0.03	0.89 ± 0.02	1.3 ± 0.7	0.68 ± 0.03	0.81 ± 0.05
2-Phenylethyl acetate	1.8 ± 0.1	2.30 ± 0.07	ND	2.08 ± 0.01	1.73 ± 0.00	ND	4.79 ± 0.01	ND	4.69 ± 0.02	2.28 ± 0.02
Methyl hydrocinnamate	0.07 ± 0.03	0.07 ± 0.01	0.3 ± 0.2	0.27 ± 0.04	0.03 ± 0.03	0.01 ± 0.01	0.52 ± 0.05	0.38 ± 0.01	0.60 ± 0.01	0.92 ± 0.09
Benzyl pentanoate	ND	ND	ND	ND	0.64 ± 0.03	0.2 ± 0.2	ND	ND	0.54 ± 0.00	0.3 ± 0.1
**Phenols ^d^**										
*o*-Guaiacol	ND	ND	42 ± 2	39.0 ± 0.3	18.6 ± 0.7	39.9 ± 0.8	49.0 ± 0.7	58 ± 9	12 ± 1	20.2 ± 0.4
*p*-Creosol	64 ± 6	78 ± 5	99 ± 6	96 ± 1	164 ± 3	185 ± 3	128 ± 2	131 ± 20	131 ± 9	128 ± 3
Phenol	0.58 ± 0.03	0.8 ± 0.1	20 ± 1	18.1 ± 0.2	3.9 ± 0.1	5.8 ± 0.4	21.2 ± 0.8	24 ± 3	12 ± 1	13.59 ± 0.07
*p*-Ethyl guaiacol	0.08 ± 0.00	0.09 ± 0.00	0.28 ± 0.04	0.30 ± 0.00	0.19 ± 0.00	0.18 ± 0.03	0.35 ± 0.03	0.30 ± 0.03	2.33 ± 0.05	0.65 ± 0.02
*p*-Cresol	0.8 ± 0.1	0.93 ± 0.04	1.9 ± 0.1	1.81 ± 0.02	2.89 ± 0.04	4.7 ± 0.1	2.85 ± 0.09	2.9 ± 0.5	2.3 ± 0.1	2.37 ± 0.01
4-Ethyl phenol	0.77 ± 0.07	0.53 ± 0.03	2.0 ± 0.2	3.40 ± 0.02	1.65 ± 0.01	12.9 ± 0.7	30 ± 1	7 ± 1	59 ± 4	43 ± 1
**Terpenes ^d^**										
Linalool	0.74 ± 0.05	0.88 ± 0.03	1.44 ± 0.03	1.63 ± 0.00	1.08 ± 0.04	1.09 ± 0.00	1.92 ± 0.04	1.9 ± 0.2	1.7 ± 0.1	1.60 ± 0.07
α-Terpineol	0.77 ± 0.06	0.70 ± 0.09	0.74 ± 0.02	0.72 ± 0.03	0.57 ± 0.00	0.71 ± 0.02	0.62 ± 0.01	0.6 ± 0.1	0.64 ± 0.03	1.89 ± 0.08
**Others ^d^**										
Dimethyl sulfide	1.4 ± 0.4	2 ± 1	1.99 ± 0.04	2.3 ± 0.2	1.7 ± 0.2	2.5 ± 0.2	1.5 ± 0.1	1.14 ± 0.04	1.5 ± 0.7	1.86 ± 0.04
Styrene	2.1 ± 0.1	4.2 ± 0.8	3.14 ± 0.05	2.4 ± 0.2	3.9 ± 0.3	4 ± 1	4.5 ± 0.2	3.6 ± 0.7	5 ± 1	4.6 ± 0.3
3-Ethylpyridine	4.3 ± 0.3	5.87 ± 0.01	2.89 ± 0.06	4.5 ± 0.2	1.7 ± 0.1	1.1 ± 0.1	2.4 ± 0.9	3.4 ± 0.7	5 ± 1	2.95 ± 0.09
3-Ethyl-4-methylpyridine	4.4 ± 0.8	6.5 ± 0.6	3.7 ± 0.6	5.5 ± 0.3	2.8 ± 0.2	4 ± 1	2.3 ± 0.3	3.0 ± 0.6	13 ± 1	6 ± 1
1,4-Dimethoxybenzene	1.8 ± 0.1	2.1 ± 0.1	2.02 ± 0.05	2.92 ± 0.08	2.2 ± 0.2	2.43 ± 0.02	3.2 ± 0.1	3.4 ± 0.5	3.4 ± 0.1	3.06 ± 0.06

^a^ Values are means ± SD. ND = not detected. See Table 5 for the meanings of samples abbreviations. ^b^ Quantitative determination expressed in g L^−1^. ^c^ Quantitative determination expressed in mg L^−1^. ^d^ Semi-quantitative determination expressed as µg L^−1^ of 3-octanol.

**Table 4:** The intensity ratings for the sensory spoilage descriptors of olive brines.

	Sample								
Descriptor	GFC1	GFL1	GCC1	GCL2	MFC2	MFL1	MCC2	MCL2	M1 (control)
Zapatera	5.7 ± 0.7 *	5.0 ± 0.6 *	4.9 ± 0.6 *	4.6 ± 0.6 *	5.3 ± 0.5 *	4.3 ± 0.5	5.0 ± 0.5 *	3.6 ± 0.4	2.4 ± 0.4
Butyric	4.5 ± 0.6 *	3.7 ± 0.6	2.7 ± 0.4	3.4 ± 0.5	3.5 ± 0.5	4.6 ± 0.7 *	2.5 ± 0.3	4.3 ± 0.6 *	2.3 ± 0.3
Putrid	2.8 ± 0.4	2.7 ± 0.5	3.0 ± 0.5	3.1 ± 0.6	2.3 ± 0.4	2.6 ± 0.5	2.0 ± 0.3	2.7 ± 0.4	1.4 ± 0.2

Values are the mean (± standard error) scores from 15 panelists with 2 replications (n = 30). For a given descriptor, an asterisk denotes that the mean value is significantly different from the control sample at the 0.05 level according to Dunnett´s *t*-test. See Table 5 for the meanings of samples abbreviations.

**Table 5:** Alkaline and washing treatments. Lye concentrations were 18.0 g L^−1^ and 19.4 g L^−1^ for Gordal (G) and Manzanilla (M) cultivars, respectively. The ambient temperature was 24 °C.

Sample ^a^	Duration (h:min)		Initial Brine (g L^−1^ NaCl)
	Lye Treatment	Washing	
G1, G2 (control)	8:10	15:00	122
GFC1, GFC2	8:30	0:50	71
GFL1, GFL2	8:30	14:00	71
GCC1, GFL2	5:00	0:50	71
GCL1, GCL2	5:00	14:00	71
M1, M2 (control)	5:55	17:20	122
MFC1, MFC2	6:05	1:05	71
MFL1, MFL2	6:05	17:05	71
MCC1, MCC2	3:15	1:05	71
MCL1, MCL2	3:15	17:05	71

^a^ In the 3-letter abbreviations, the first letter refers to the cultivar (G, M), the second one refers to the alkaline treatment (F and C stand for forceful and curtailed, respectively), and the third one refers to the washing step (L and C stand for long and curtailed, respectively).

## Supplementary Materials

The following are available online at http://www.mdpi.com/2218-1989/8/4/73/s1. Table S1: Number of reads and genus diversity estimators for 16s rRNA amplicons from Manzanilla and Gordal samples at the post-fermentation stage; Table S2: Number of reads and genus diversity estimators for ITS amplicons from Manzanilla and Gordal samples at the post-fermentation stage; Table S3: Pearson´s correlation coefficients between the mean values of the relative abundance of microorganisms and the sensory spoilage descriptors; Table S4: Pearson´s correlation coefficients between bacterial communities and metabolites; Table S5: Pearson´s correlation coefficients between yeast communities and metabolites; Figure S1: Concentrations of lactic and acetic acids in brine samples after 7 and 11 months of brining. Error bars denote standard deviations of triplicate analyses; Figure S2: Total contents of volatile acids and phenols in brine samples. Error bars indicate 95% confidence intervals; Figure S3: Dendrogram of (a) the observations (samples), and (b) variables (metabolites) from the chemical data obtained from agglomerative hierarchical cluster (AHC) analysis; Figure S4: Plot of VIPs (sorted in descending order) with jack-knife uncertainty bars (95%) from the PLS regression between: (a) volatile metabolites and butyric descriptor, and (b) volatile metabolites and zapatera descriptor.
