# Supplementary material for: Microbiota and Metabolite Profiling of Spoiled Spanish-Style Green Table Olives
Source: Metabolites. 2018 Oct 31;8(4):73. doi: 10.3390/metabo8040073 (PMC6316098; doi:10.3390/metabo8040073)
Supplement: Supplementary file 1 [file metabolites-08-00073-s001.pdf]

Table S1. Number of reads and genus diversity estimators for 16s rRNA amplicons from Manzanilla and Gordal samples at the post-fermentation stage.

Sample[a]	Reads (bp)	MeanLength (bp)	Shannon	Simpson	CHAO1
G (control)	66750	464.78	0.95	0.56	15
GCC1	63439	457.07	0.83	0.38	28
GCC2	116975	460.60	1.12	0.58	35
GCL1	130128	461.06	0.80	0.40	39
GCL2	61370	463.93	0.64	0.28	32
GFC1	41505	463.93	1.19	0.57	26
GFC2	41278	464.60	1.05	0.52	23
GFL1	52341	464.60	0.70	0.31	34
GFL2	56775	464.71	0.83	0.37	25
M (control)	29930	441.81	0.83	0.42	29
MCC1	76358	460.04	0.80	0.50	16
MCC2	81597	461.15	0.99	0.54	33
MCL1	82283	461.34	1.29	0.54	42
MCL2	86826	460.66	1.00	0.42	41
MFC1	97273	443.69	1.12	0.59	47
MFC2	91625	459.52	1.08	0.55	51
MFL1	69618	462.78	1.11	0.48	46
MFL2	35337	458.70	0.78	0.32	19

[a] Results of G and M controls correspond to the blend of both duplicates. See Table 5 for the meanings of samples abbreviations.

Table S2. Number of reads and genus diversity estimators for ITS amplicons from Manzanilla and Gordal samples at the post-fermentation stage.

Sample[a]	Reads (bp)	MeanLength (bp)	Shannon	Simpson	CHAO1
G (control)	48015	505.97	0.48	0.27	1
GCC1	52714	426.93	0.01	0.00	13
GCC2	49660	505.52	0.01	0.00	11
GCL1	66403	505.45	0.05	0.02	16
GCL2	52897	506.16	0.06	0.02	17
GFC1	54532	497.78	0.07	0.02	3
GFC2	36573	502.23	0.03	0.01	15
GFL1	25600	505.76	0.43	0.18	15
GFL2	33803	436.65	0.23	0.08	16
M (control)	32310	510.80	0.19	0.07	1
MCC1	39671	499.18	0.48	0.25	17
MCC2	33530	502.70	0.02	0.00	18
MCL1	33559	493.71	0.47	0.22	15
MCL2	44605	506.16	0.41	0.18	17
MFC1	64996	510.06	0.23	0.08	4
MFC2	42917	505.74	0.64	0.31	17
MFL1	51464	487.30	0.58	0.29	17
MFL2	63070	505.49	0.65	0.33	18

[a] Results of G and M controls correspond to the blend of both duplicates. See Table 5 for the meanings of samples abbreviations.

Table S3. Pearson´s correlation coefficients between the mean values of the relative abundance of microorganisms and the sensory spoilage descriptors[a]

Microorganisms	Zapatera	Butyric
Genus *Corynebacterium*	0.056	0.534
Genus *Propionibacterium*	0.750*	0.468
Unclassified *Bacillaceae*	-0.874**	-0.475
Genus *Natronobacillus*	-0.839**	-0.365
Genus *Oceanobacillus*	-0.957***	-0.175
Genus *Lactobacillus*	-0.630	-0.677*
Genus *Ruminococcus*	0.581	0.734*
Family *Cardiobacteriaceae*	0.547	0.430
Family *Enterobacteriaceae*	-0.374	0.435
Candida apicola	-0.536	-0.405
Candida etchellsii	-0.767*	-0.510
Candida pararugosa	-0.142	0.410
Dekkera bruxellensis	-0.319	0.307
Family *Dipodascaceae*	0.158	0.441
Pichia manshurica	0.169	0.074
Pichia membranifaciens	0.009	-0.496

[a] Asterisks denote statistical significance at $p < 0.05$ (*), $p < 0.01$ (**), and $p < 0.001$ (***).

Table S4. Pearson´s correlation coefficients between bacterial communities and metabolites[a]

Variables	*Corynebacterium*	*Propionibacterium*	*Bacillaceae*	*Natronobacillus*	*Oceanobacillus*	*Lactobacillus*	*Ruminococcus*	*Cardiobacteriaceae*	*Enterobacteriaceae*
Lactic acid	-0.368	**-0.509***	**0.835*****	**0.725****	**0.653****	**0.853*****	**-0.604****	**-0.738*****	-0.151
Succinic acid	-0.123	**0.477***	-0.318	**-0.490***	-0.197	-0.056	0.264	-0.054	0.123
Acetic acid	0.025	**0.659****	**-0.511***	-0.281	**-0.576***	-0.032	0.218	-0.044	-0.363
Propionic acid	0.067	**0.687****	**-0.706****	**-0.522***	**-0.692****	-0.366	0.462	0.251	-0.279
Butyric acid	**0.532***	0.375	-0.317	-0.127	-0.284	**-0.689****	0.452	**0.543***	0.052
Isobutyric acid	-0.259	-0.187	-0.050	-0.133	-0.175	-0.051	0.031	0.128	-0.306
Valeric acid	0.433	0.290	-0.329	-0.167	-0.317	**-0.710****	0.440	**0.596****	0.036
Caproic acid	0.271	0.192	-0.244	-0.126	-0.310	**-0.514***	0.257	**0.477***	-0.069
Heptanoic acid	0.030	0.102	-0.197	-0.153	-0.294	-0.354	0.181	0.356	-0.117
Cyclohexanoic acid	-0.196	0.286	-0.354	-0.372	-0.221	-0.312	0.286	0.267	0.076
Ethanol	-0.220	-0.001	-0.238	-0.284	-0.287	0.212	-0.326	-0.025	-0.124
2-Butanol	**0.630****	-0.106	-0.032	-0.074	0.301	-0.359	0.094	0.260	**0.963*****
1-Propanol	**-0.794*****	-0.129	0.221	-0.023	-0.021	0.430	-0.206	-0.293	**-0.608****
1-Butanol	-0.330	0.103	-0.277	-0.327	-0.359	-0.348	0.373	0.321	-0.330
1-Pentanol	0.003	0.108	-0.286	-0.235	-0.362	**-0.483***	0.411	0.412	-0.120
3-Methyl-1-pentanol	-0.334	0.026	-0.295	-0.391	-0.430	0.099	-0.120	0.045	-0.312
1-Hexanol	-0.030	0.034	-0.160	-0.105	-0.319	-0.251	0.109	0.284	-0.218
(*Z*)-3-Hexen-1-ol	-0.103	-0.003	0.050	0.210	0.023	**0.607****	-0.339	**-0.560***	-0.027
2-Ethyl-1-hexanol	-0.359	-0.300	-0.154	-0.226	-0.180	-0.320	0.089	0.441	-0.185
1-Octanol	0.105	0.073	**-0.571***	**-0.539***	**-0.613****	-0.444	0.146	**0.516***	0.091
Benzyl alcohol	0.143	0.311	-0.369	-0.213	-0.282	-0.026	-0.103	0.074	0.099
Phenylethyl alcohol	-0.140	0.135	-0.262	-0.243	-0.266	0.102	-0.172	0.005	-0.045
Octanal	0.296	-0.073	-0.180	0.019	-0.247	-0.282	0.155	0.256	0.031
Nonanal	0.421	-0.068	-0.070	0.156	-0.304	-0.269	0.001	0.298	-0.085
Benzaldehyde	0.228	0.292	-0.384	-0.246	-0.405	-0.234	0.185	0.201	-0.158
3,4-Dimethylbenzaldehyde	-0.315	-0.401	**0.873*****	**0.752*****	**0.695****	**0.765*****	**-0.506***	**-0.696****	-0.210

Methyl propanoate	-0.360	**0.485***	-0.265	-0.253	-0.429	0.217	0.188	-0.289	-0.441
Propyl acetate	-0.335	-0.005	-0.106	-0.135	-0.248	**0.474***	-0.341	-0.319	-0.337
Methyl 2-methylbutanoate	-0.031	-0.196	0.253	0.329	0.039	0.322	-0.052	-0.348	-0.307
Ethyl butanoate	0.085	0.325	-0.118	-0.172	0.043	-0.244	0.033	0.231	0.257
Propyl propanoate	**-0.545***	0.318	-0.213	-0.395	-0.352	0.273	-0.022	-0.211	**-0.516***
Methyl pentanoate	0.394	-0.009	-0.356	-0.256	-0.224	**-0.777*****	0.323	**0.751*****	0.313
Ethyl pentanoate	-0.250	-0.175	-0.177	-0.201	-0.102	-0.267	-0.077	0.427	-0.069
Methyl hexanoate	0.401	-0.006	-0.227	-0.160	-0.122	**-0.569***	0.130	**0.578***	0.325
Propyl pentanoate	-0.263	0.085	-0.253	-0.284	-0.185	-0.356	0.074	0.451	-0.128
Ethyl hexanoate	0.429	-0.003	-0.011	-0.092	0.268	-0.204	0.028	0.128	**0.778*****
Methyl heptanoate	-0.048	-0.046	-0.149	-0.135	-0.252	-0.261	0.059	0.321	-0.115
Propyl hexanoate	-0.192	0.002	-0.175	-0.197	-0.097	-0.274	-0.074	0.412	-0.086
Methyl cyclohexanecarboxylate	-0.221	0.083	-0.322	-0.343	-0.183	-0.241	0.218	0.232	0.091
Ethyl cyclohexanecarboxylate	-0.189	-0.038	-0.286	-0.323	-0.155	-0.278	0.093	0.340	0.117
Benzyl propanoate	-0.100	0.403	**-0.574***	-0.443	**-0.481***	-0.112	0.125	0.123	-0.072
2-Phenylethyl acetate	-0.150	-0.051	-0.131	-0.194	0.014	-0.049	0.099	0.024	0.307
Methyl hydrocinnamate	-0.254	-0.169	-0.329	-0.376	-0.174	-0.266	-0.026	0.406	0.158
Benzyl pentanoate	**0.561***	0.419	-0.180	-0.083	0.058	**-0.472***	0.289	0.308	0.426
o-Guaiacol	0.012	0.107	-0.382	-0.266	-0.442	0.003	-0.159	0.128	-0.155
p-Creosol	0.359	0.200	**-0.590***	-0.405	**-0.582***	**-0.744*****	0.305	**0.760*****	-0.007
Phenol	-0.043	0.112	-0.446	-0.376	-0.390	0.022	-0.173	0.109	0.042
p-Ethyl guaiacol	0.468	0.068	-0.099	-0.187	0.202	-0.254	0.146	0.125	**0.873*****
p-Cresol	**0.619****	0.059	-0.434	-0.184	**-0.500***	-0.443	-0.016	**0.517***	0.100
4-Ethyl phenol	0.428	-0.132	-0.107	-0.193	0.188	-0.214	-0.019	0.183	**0.893*****
Linalool	**0.508***	0.012	-0.095	0.109	0.029	0.184	-0.224	-0.190	0.435
α-Terpineol	**-0.470***	0.192	-0.247	-0.415	-0.284	-0.340	0.322	0.340	-0.295
Dimethyl sulfide	0.357	-0.019	0.097	0.382	0.036	0.015	-0.004	-0.083	0.085
Styrene	-0.055	-0.219	-0.097	-0.122	0.035	-0.076	-0.185	0.212	0.109
3-Ethylpyridine	0.234	-0.079	0.442	**0.498***	**0.544***	**0.524***	-0.209	**-0.643****	**0.470***

3-Ethyl-4-methylpyridine	**0.576***	0.093	0.166	0.226	0.410	0.025	0.067	-0.224	**0.843*** **
1,4-Dimethoxybenzene	0.397	0.035	-0.246	-0.219	-0.064	-0.063	-0.101	0.075	**0.541***

[a]Values in bold are different from 0. Asterisks denote significance at $p < 0.05$ (*), $p < 0.01$ (**), and $p < 0.001$ (***).

Table S5. Pearson´s correlation coefficients between yeast communities and metabolites[a]

Variables	*Candida apicola*	*Candida etchellsii*	*Candida pararugosa*	*Dekkera bruxellensis*	*Dipodascaceae*	*Pichia manshurica*	*Pichia membranifaciens*
Lactic acid	**0.703****	**0.667****	-0.160	0.094	-0.276	-0.079	-0.143
Succinic acid	-0.239	-0.084	0.261	**-0.525***	-0.363	0.041	0.347
Ethanol	-0.183	0.250	-0.192	-0.205	-0.434	-0.090	0.311
Acetic acid	-0.030	-0.193	-0.323	-0.084	0.057	-0.140	0.157
Propionic acid	-0.326	**-0.473***	-0.116	-0.175	0.023	-0.050	0.325
Butyric acid	-0.333	-0.441	0.068	0.351	**0.522***	0.215	-0.239
Isobutyric acid	-0.165	0.303	-0.062	0.132	-0.148	-0.212	-0.023
Valeric acid	-0.360	-0.446	0.262	0.229	0.448	0.225	-0.210
Caproic acid	-0.269	-0.294	0.347	0.133	0.352	0.159	-0.235
Heptanoic acid	-0.184	-0.189	0.464	-0.059	0.153	0.057	-0.136
Cyclohexanoic acid	-0.139	-0.360	0.001	-0.170	-0.128	-0.249	0.373
2-Butanol	-0.277	-0.230	0.314	-0.059	0.358	-0.108	-0.259
1-Propanol	0.260	0.468	-0.202	-0.292	**-0.704****	0.277	0.405
1-Butanol	-0.189	-0.099	0.302	-0.062	-0.210	0.281	0.184
1-Pentanol	-0.207	-0.292	0.406	-0.082	0.162	0.109	-0.065
3-Methyl-1-pentanol	-0.264	0.400	-0.022	-0.296	-0.408	0.080	0.144
1-Hexanol	-0.092	-0.095	0.357	-0.063	0.145	0.078	-0.166
(*Z*)-3-Hexen-1-ol	0.427	0.206	-0.170	-0.121	-0.080	-0.240	-0.013
2-Ethyl-1-hexanol	-0.203	-0.170	0.236	-0.056	-0.287	0.430	0.312
1-Octanol	**-0.562***	-0.138	0.099	-0.354	0.007	-0.128	0.081
Benzyl alcohol	-0.221	-0.203	-0.199	-0.093	-0.068	-0.216	0.261
Phenylethyl alcohol	-0.201	-0.014	-0.240	-0.230	-0.352	-0.304	0.427
Octanal	-0.155	-0.253	-0.312	0.130	0.335	-0.140	-0.073
Nonanal	-0.101	-0.266	-0.177	-0.019	**0.566***	-0.205	-0.318

Benzaldehyde	-0.282	-0.418	-0.432	-0.074	0.151	0.186	0.228
3,4-Dimethylbenzaldehyde	**0.657****	**0.497***	-0.262	0.196	-0.283	-0.097	0.001
Methyl propanoate	0.061	0.056	-0.056	-0.465	-0.242	-0.201	0.222
Propyl acetate	0.178	**0.489***	-0.213	-0.253	-0.197	-0.072	-0.072
Methyl 2-methylbutanoate	0.322	0.314	0.133	0.058	0.203	0.106	-0.444
Ethyl butanoate	-0.102	-0.177	-0.061	0.079	-0.075	-0.126	0.208
Propyl propanoate	0.015	0.343	-0.267	-0.394	-0.461	0.058	0.286
Methyl pentanoate	**-0.475***	**-0.506***	0.295	0.214	0.396	0.013	-0.116
Ethyl pentanoate	-0.185	-0.175	0.072	0.163	-0.246	-0.092	0.341
Methyl hexanoate	-0.351	-0.332	0.290	0.218	0.402	0.014	-0.235
Propyl pentanoate	-0.194	-0.254	-0.022	0.094	-0.283	0.099	0.441
Ethyl hexanoate	-0.151	-0.124	-0.077	-0.001	0.234	-0.107	-0.117
Methyl heptanoate	-0.154	-0.124	**0.499***	-0.110	0.111	0.072	-0.147
Propyl hexanoate	-0.154	-0.186	-0.011	0.190	-0.211	0.034	0.326
Methyl cyclohexanecarboxylate	-0.190	-0.330	-0.021	-0.148	-0.151	-0.222	0.381
Ethyl cyclohexanecarboxylate	-0.195	-0.302	-0.113	-0.055	-0.137	-0.191	0.370
Benzyl propanoate	-0.282	-0.286	-0.119	-0.218	-0.163	-0.219	0.391
2-Phenylethyl acetate	-0.092	0.107	-0.107	-0.102	-0.171	-0.193	0.134
Methyl hydrocinnamate	-0.332	-0.208	0.136	-0.116	-0.337	-0.192	0.445
Benzyl pentanoate	-0.260	-0.300	0.347	0.318	0.353	0.261	-0.239
o-Guaiacol	-0.270	-0.083	-0.233	-0.189	-0.004	-0.270	0.137
p-Creosol	**-0.611****	-0.460	0.226	0.057	0.269	0.243	-0.014
Phenol	-0.260	-0.059	-0.079	-0.290	-0.127	-0.273	0.203
p-Ethyl guaiacol	-0.235	-0.153	-0.001	-0.160	0.208	-0.138	-0.088
p-Cresol	**-0.478***	-0.244	0.106	0.038	**0.566***	-0.151	-0.392
4-Ethyl phenol	-0.290	0.025	0.320	-0.221	0.165	-0.182	-0.235
linalool	0.076	0.054	0.111	0.009	0.413	-0.212	-0.450
α-Terpineol	-0.281	-0.190	-0.027	-0.237	**-0.491***	0.126	**0.603****
Dimethyl sulfide	0.362	-0.098	0.115	0.116	**0.646****	0.051	**-0.598****

Styrene	-0.234	-0.072	-0.018	0.146	-0.099	-0.024	0.152
3-Ethylpyridine	**0.617****	0.121	-0.066	0.001	0.246	-0.270	-0.302
3-Ethyl-4-methylpyridine	0.201	-0.142	0.052	-0.047	0.430	-0.234	-0.329
1,4-Dimethoxybenzene	-0.173	-0.041	0.023	-0.200	0.206	-0.206	-0.161

[a]Values in bold are different from 0. Asterisks denote significance at $p < 0.05$ (*), $p < 0.01$ (**), and $p < 0.001$ (***).

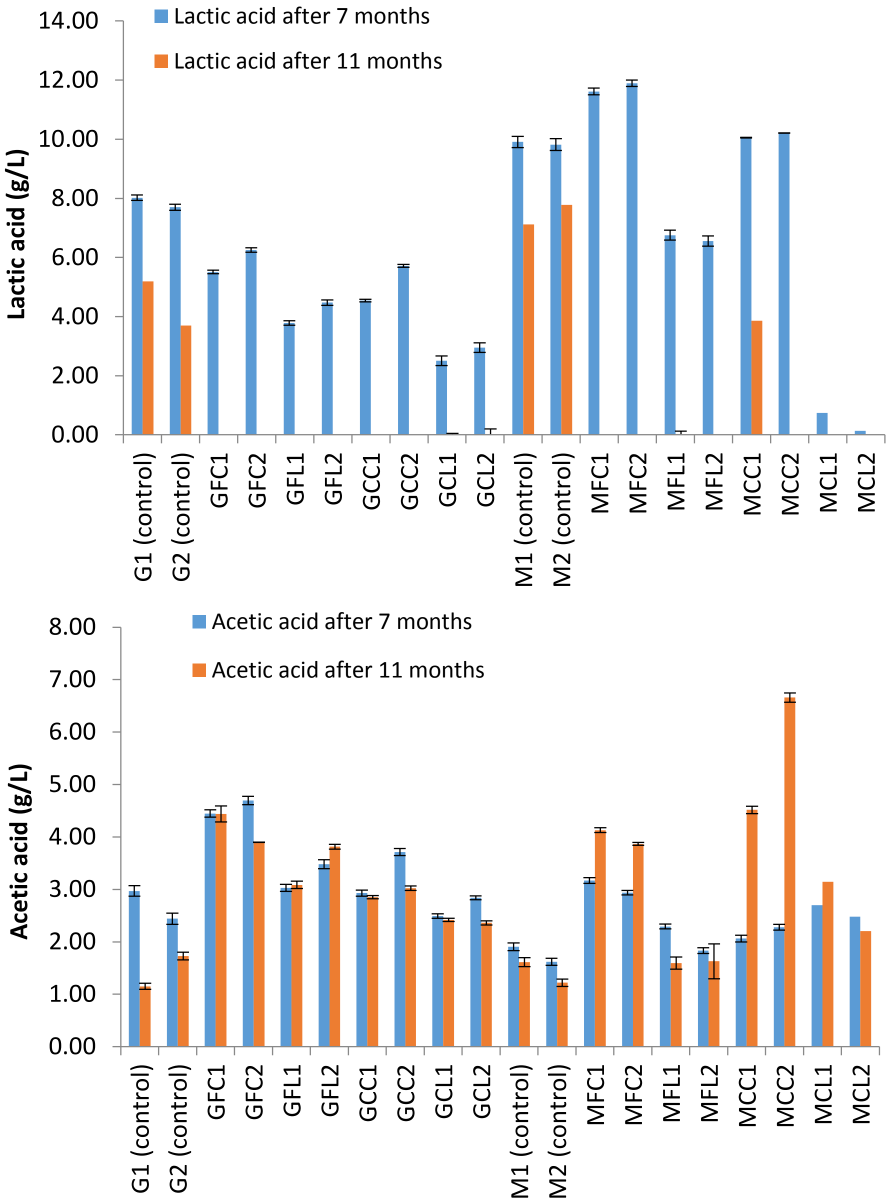

Figure S1. Concentrations of lactic and acetic acids in brine samples after 7 and 11 months of brining. Error bars denote standard deviations of triplicate analyses. See Table 5 for the meanings of samples abbreviations.

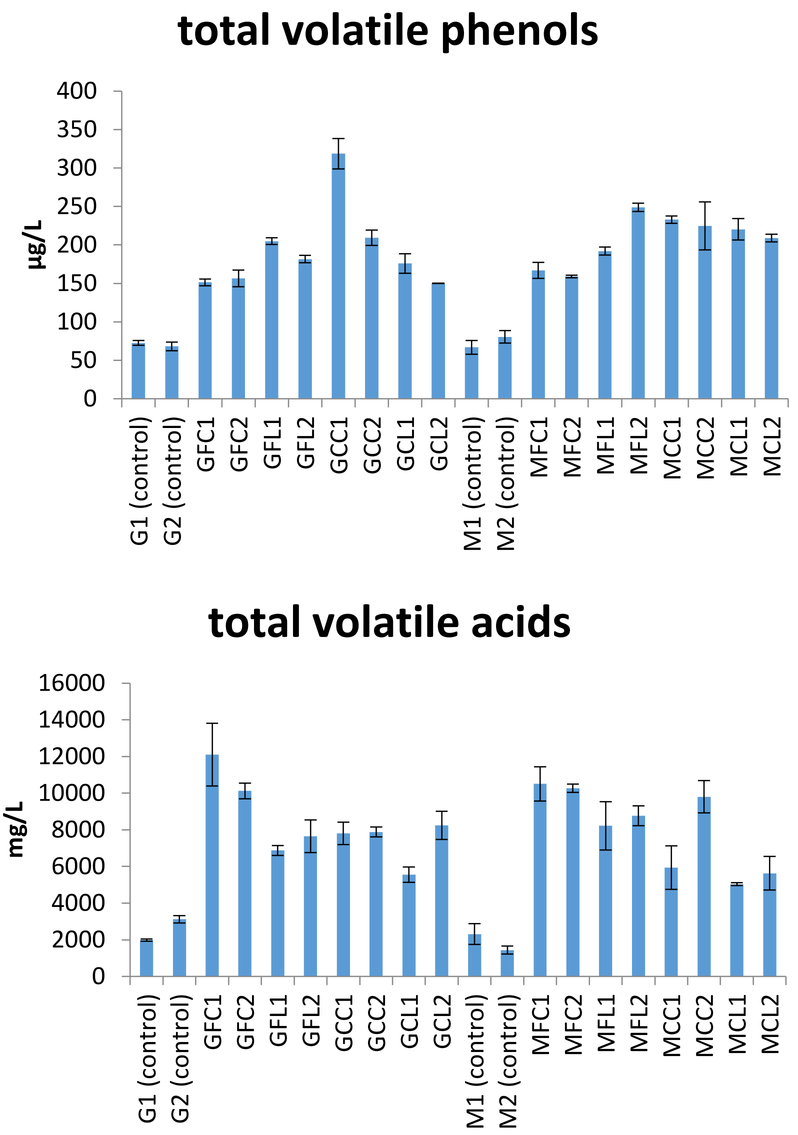

Figure S2. Total contents of volatile acids and phenols in brine samples. Error bars indicate 95% confidence intervals. See Table 5 for the meanings of samples abbreviations.

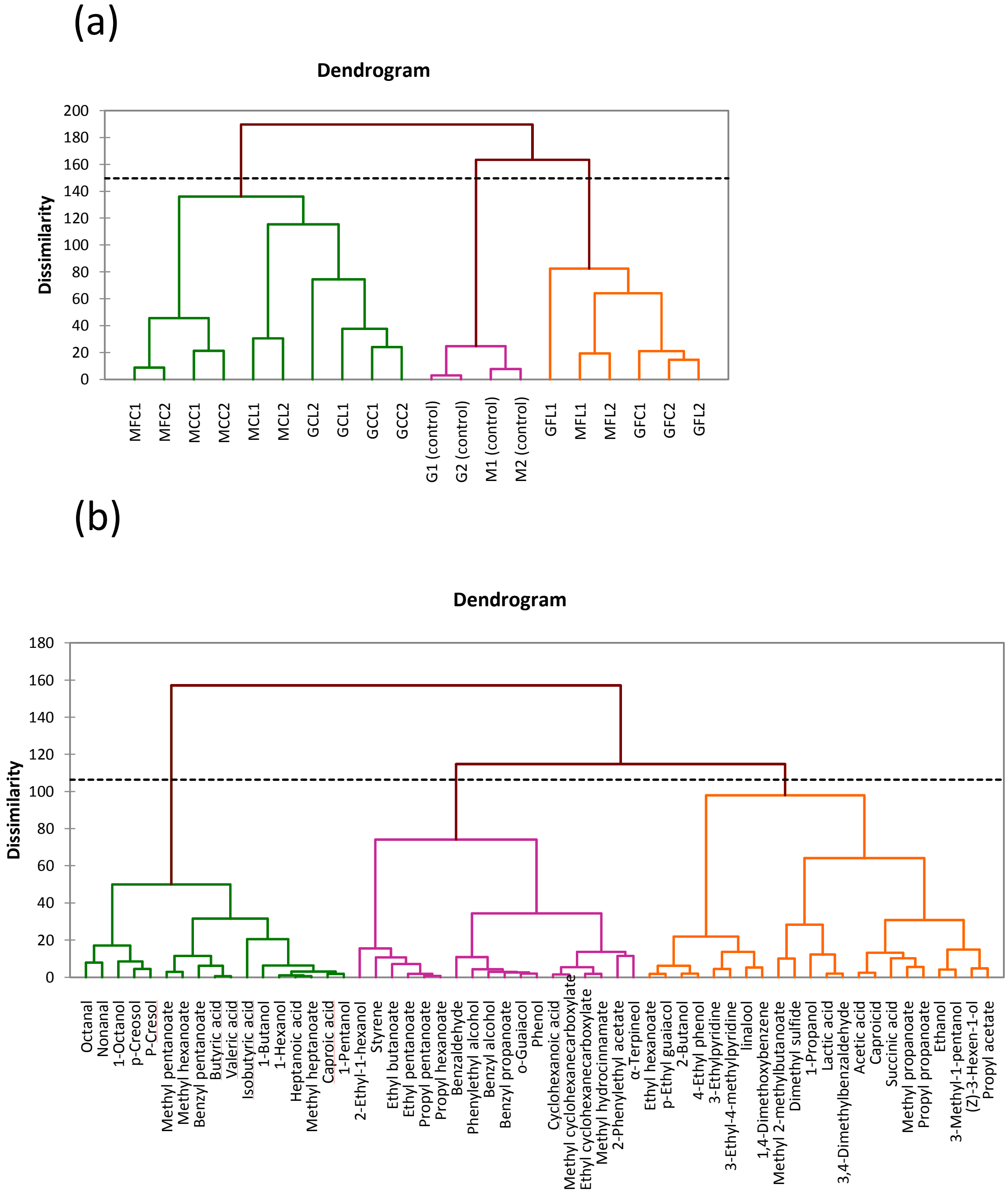

Figure S3. Dendrogram of (a) the observations (samples), and (b) variables (metabolites) from chemical data obtained by agglomerative hierarchical cluster (AHC) analysis. See Table 5 for the meanings of samples abbreviations.

(a)

(b)

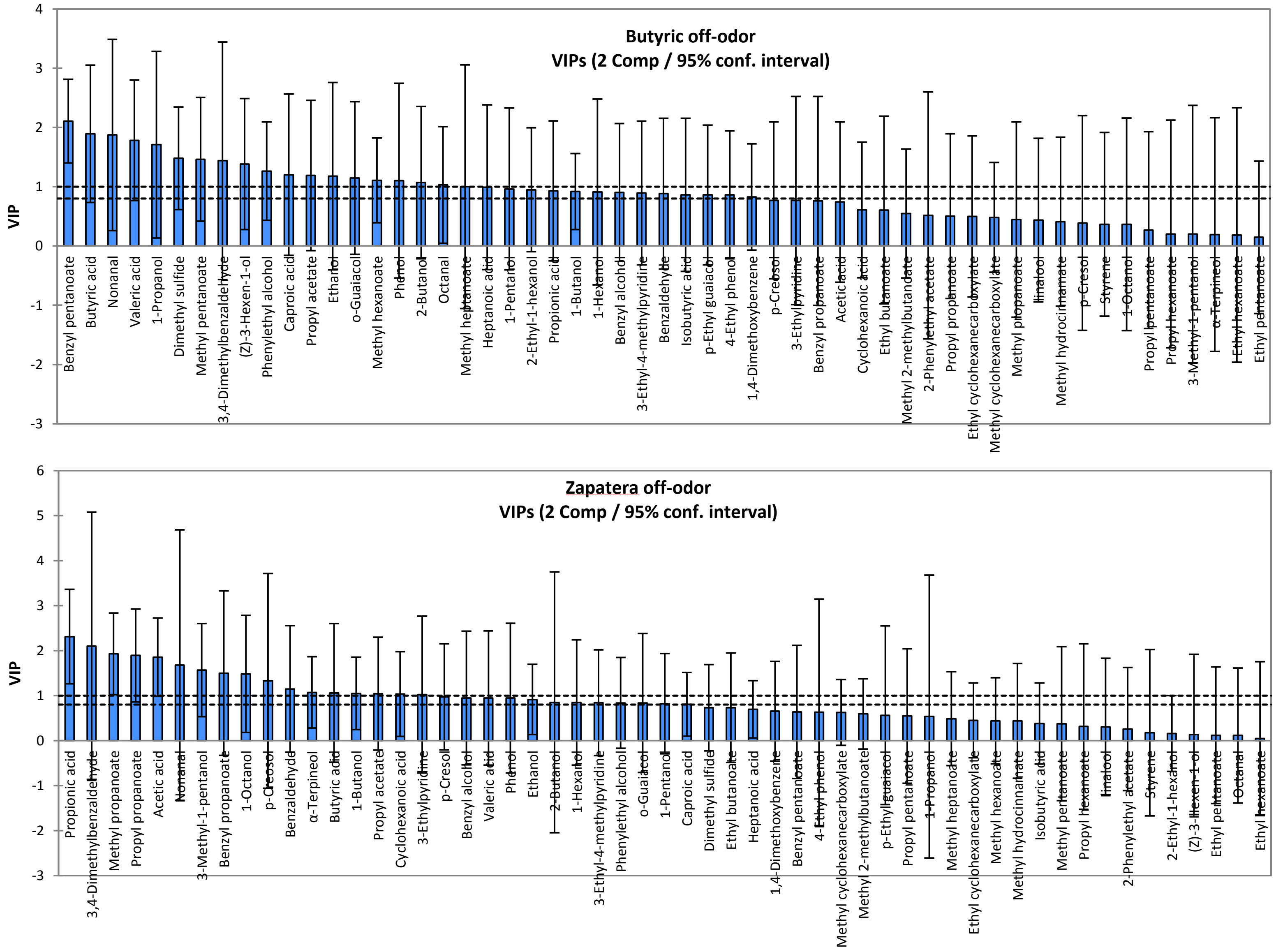

Figure S4. Plot of VIPs (sorted in descending order) with jack-knife uncertainty bars (95%) from the PLS regression between: (a) volatile metabolites and butyric descriptor, and (b) volatile metabolites and zapatera descriptor.
